# Robotic-Assisted vs. Standard Laparoscopic Surgery for Rectal Cancer Resection: A Systematic Review and Meta-Analysis of 19,731 Patients

**DOI:** 10.3390/cancers14010180

**Published:** 2021-12-30

**Authors:** Kamil Safiejko, Radoslaw Tarkowski, Maciej Koselak, Marcin Juchimiuk, Aleksander Tarasik, Michal Pruc, Jacek Smereka, Lukasz Szarpak

**Affiliations:** 1 Colorectal Cancer Unit, Maria Sklodowska-Curie Bialystok Oncology Center, 15-027 Bialystok, Poland; kamil.safiejko@gmail.com (K.S.); jumedica.onkologia@gmail.com (M.J.); olek.tarasik@gmail.com (A.T.); 2 Department of Surgical Oncology, Regional Specialist Hospital, 55-220 Legnica, Poland; rt@rakpiersi.net; 3 Institute of Outcomes Research, Maria Sklodowska-Curie Medical Academy, 03-411 Warsaw, Poland; mkoselak@wp.pl; 4 Oncological Surgery Subdivision, Masovian Oncology Hospital, 05-135 Legionowo, Poland; 5 Research Unit, Polish Society of Disaster Medicine, 05-806 Warsaw, Poland; m.pruc@ptmk.org (M.P.); Jacek.Smereka@umed.wroc.pl (J.S.); 6 Department of Emergency Medical Service, Wroclaw Medical University, 52-443 Wroclaw, Poland; 7 Henry JN Taub Department of Emergency Medicine, Baylor College of Medicine, Houston, TX 77030, USA; 8 Research Unit, Maria Sklodowska-Curie Bialystok Oncology Center, 15-027 Bialystok, Poland

**Keywords:** rectal cancer, robotic-assisted laparoscopic, conventional laparoscopic surgery, outcome, systematic review, meta-analysis

## Abstract

**Simple Summary:**

Surgery remains a mainstay of combined modality treatment at patients with rectal cancer; however, there is a growing interest in using laparoscopic techniques (LG); including robotic-assisted techniques (RG). Therefore, we have prepared a meta-analysis of the literature regarding the safety and efficacy of robotic versus laparoscopic approaches in patients undergoing curative surgery for rectal cancer. The results indicate a number of advantages of RG in terms of both safety and efficacy. Operative time in the RG group was shorter and associated with a statistically significantly lower conversion of the procedure to open surgery. RG technique provided a shorter duration of hospital stay and lowered urinary risk retention. No differences were found between these techniques regarding TNM stage; N stage or lymph nodes harvested. Survival to hospital discharge or 30-day overall survival rate was 99.6% in RG vs. 98.8% for LG.

**Abstract:**

Robotic-assisted surgery is expected to have advantages over standard laparoscopic approach in patients undergoing curative surgery for rectal cancer. PubMed, Cochrane Library, Web of Science, Scopus and Google Scholar were searched from database inception to 10 November 2021, for both RCTs and observational studies comparing robotic-assisted versus standard laparoscopic surgery for rectal cancer resection. Where possible, data were pooled using random effects meta-analysis. Forty-Two were considered eligible for the meta-analysis. Survival to hospital discharge or 30-day overall survival rate was 99.6% for RG and 98.8% for LG (OR = 2.10; 95% CI: 1.00 to 4.43; *p* = 0.05). Time to first flatus in the RG group was 2.5 ± 1.4 days and was statistically significantly shorter than in LG group (2.9 ± 2.0 days; MD = −0.34; 95%CI: −0.65 to 0.03; *p* = 0.03). In the case of time to a liquid diet, solid diet and bowel movement, the analysis showed no statistically significant differences (*p* > 0.05). Length of hospital stay in the RG vs. LG group varied and amounted to 8.0 ± 5.3 vs. 9.5 ± 10.0 days (MD = −2.01; 95%CI: −2.90 to −1.11; *p* < 0.001). Overall, 30-days complications in the RG and LG groups were 27.2% and 19.0% (OR = 1.11; 95%CI: 0.80 to 1.55; *p* = 0.53), respectively. In summary, robotic-assisted techniques provide several advantages over laparoscopic techniques in reducing operative time, significantly lowering conversion of the procedure to open surgery, shortening the duration of hospital stay, lowering the risk of urinary retention, improving survival to hospital discharge or 30-day overall survival rate.

## 1. Introduction

Colorectal cancer remains the second most common cause of death in the Western world, and rectal localization accounts for approximately 25% of its cases. Surgery remains a mainstay of combined modality treatment at patients with rectal cancer. Total Mesorectal Excision (TME) proposed by Heald et al. [1] became a golden standard, improving both surgical radicalness of cancer eradication and quality of life due to hypogastric nerves preservation and its impact on urinary avoidance and sexual functions. Direct vision enabled by St Marks retractors and more extended tools allow one to resect mesorectum within the intact fascia, obtain a proper circumferential margin (CRM) and thus better oncologic radicalness through eradicating cancer deposits localized within mesorectum, resected en bloc together with and affected organ. This has been the opposite for former blunt resections performed directly with an unarmed surgeon hand, leaving part of the structures mentioned above with cancer cells within the pelvis as the gateway to local recurrence.

Furthermore, the adoption of laparoscopic TME enabled equal or, in some aspects, superior results compared to open surgery. Those are lower CRM positivity rates at patients with tumours of the lower third part of the rectum, as shown in effects of the COLOR II trial [2]. The oncologic safety of laparoscopy, equal to open surgery, has been shown in different studies. The laparoscopic approach was superior to open surgery in terms of lower pain, faster recovery, shorter hospital stay and better cosmesis [3,4].

However, some substantial difficulties are present, especially during the operations at patients with tumours of the lower rectum. Those are: problems with obtaining a good view with a rigid optical system in the narrow pelvis, difficult maneuvering with long and rigid laparoscopic tools with their lack of flexibility and the hand and tool tremor, even with minimal or a loss of tactile sensation. As mentioned above, several critical organs are localized in close proximity to the narrow space of the pelvis, with access even more difficult in males and obese patients. The robotic-assisted approach was therefore accepted into the surgical armamentarium.

In 2006 Pigazzi et al. described a robot-assisted laparoscopic approach to TME. Its introduction enables potential omitting difficulties mentioned above through better 3D vision, wristed instruments enabling a higher range of maneuverability in the narrow pelvis, tremor’s abolition [5]. Robotic surgery in the treatment of rectal cancer patients has been endorsed like laparoscopy had been before, sharing the same principles but with other, improved tools overcoming aforementioned problems. Although some positive short-term aspects of robotic surgery superior to the laparoscopic approach were shown, there are no long-term outcomes proven in clinical trials. Finally, there comes an issue of cost-effectiveness of robotic surgery, expensive and with some disparities in reimbursement across different health care systems.

Therefore, we aimed to systematically assess the available evidence in the literature regarding the safety and efficacy of robotic versus laparoscopic approach in patients undergoing curative surgery for rectal cancer.

## 2. Materials and Methods

This systematic review and meta-analysis was done according to the Preferred Reporting Items for Systematic Reviews and Meta-analysis (PRISMA) statement [6] (Table S1). The study protocol has been deposited in the PROSPERO database prior to the start of the study. No protocol changes were made during the study. All analyses were based on previously published studies; thus, ethical approval or patient consent was not suitable for this meta-analysis.

### 2.1. Literature Search and Selection

Comprehensive systematic searches of online electronic databases, including PubMed, Cochrane Library, Web of Science, Scopus and Google Scholar from databases inception to November 10th, 2021, were performed. We searched the literature using the following keywords: “rectal cancer*” OR “rectal adenocarcinoma” OR “rectal tumor” OR “rectal neoplasms” AND “robotic” OR “laparoscopic” AND “surgery” OR “resection”. All records were searched by two researchers (M.P. and K.S.) separately. The decision to include or exclude a study was also made by two independent researchers. Disagreements were solved through discussion with third researcher (L.S.). The search of databases was restricted to English publications. No limitation was set for age of participants in the searched articles. We also manually checked the reference lists in each involved publication to identify eligible studies.

Studies that were included in this meta-analysis had to fulfill the following PICOS criteria: (1) Adult patients who were diagnosed with rectal and were treated with rectal cancer surgery; (2) Intervention, robotic-assisted rectal cancer surgery; (3) Comparison, laparoscopic rectal cancer surgery; (4) Outcomes, detailed information for survival or mortality; (5) Study design, randomized controlled trials comparing robotic-assisted vs. standard laparoscopic surgery for rectal cancer resection. Studies were excluded if: (1) don’t present comparator group; (2) literatures are reviews, conference articles, editorial, letters or duplicated publications.

### 2.2. Data Extraction and Quality Assessment

All the following information was separately extracted by two researchers (K.S. and L.S.): first author name, year of publication, region of cohort, patient characteristics (i.e., no. of patients, age, sex), intraoperative data (i.e., operative time, blood loss, conversion to open rate), tumor pathological data (i.e., TNM stage, lymph nodes harvested, tumor size) or postoperative outcomes (survival rate, disease free-survival rate, length of hospital stay; adverse event types). Discrepancies were resolved through discussion with the third researcher (J.S.). Data from included studies were recorded using a Microsoft Excel (Microsoft Corporation, Redmond, WA, USA) specific predefined report form. When data about the primary outcomes were missing, we planned to contact the corresponding author of the original study.

We compared data items, outcomes, design strengths and weaknesses across the studies. For each study, the risk of bias was assessed at the study level using the Rob2 tool for randomized [7] trials and ROBINS-I bias assessment tool for non-randomized studies [8]. The Robvis application was used to visualize risk of bias assessments [9].

### 2.3. Statistical Analysis

For dichotomous data, we used odds ratios (OR) as the effect measure with 95% confidence intervals (CIs) and for continuous data we used mean differences (MD) with 95% CI. When the continuous outcome was reported in a study as median, range, and interquartile range, we estimated means and standard deviations using the formula described by Hozo et al. [10]. Heterogeneity was assessed statistically using I^2^ (no heterogeneity, I^2^ =  0–25%; moderate heterogeneity, I^2^ =  25–50%; large heterogeneity, I^2^ =  50–75%; extreme heterogeneity, I^2^ = 75–100%). The random effects model was used for analyses [11]. All analyses were performed with the Review Manager software version 5.4 (Nordic Cochrane Centre, Cochrane Collaboration), and Stata software, version 15.0 (College Station, TX, USA). The significance level for all statistical tests was *p*  <  0.05 (two-tailed).

## 3. Results

### 3.1. Eligible Studies and Study Characteristics

**Figure 1:**
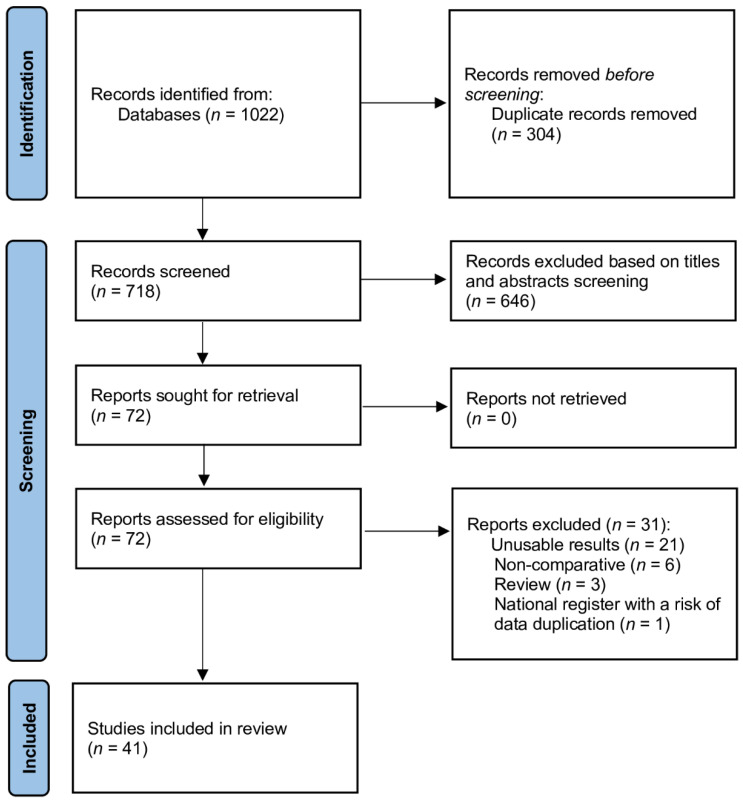
Database search and selection of studies according to PRISMA guidelines.

The literature search process identified 1022 studies (Figure 1). After excluding duplicate publications, reviews, meta-analyses, editorials, letters, abstracts and case reports, 72 studies were fully reviewed, and 41 were considered eligible for the meta-analysis [12,13,14,15,16,17,18,19,20,21,22,23,24,25,26,27,28,29,30,31,32,33,34,35,36,37,38,39,40,41,42,43,44,45,46,47,48,49,50,51]. The risk of bias according to the authors of the present study was low for 32 studies, moderate for 10 studies (Figures S1–S4).

### 3.2. Patient Characteristics

Detailed characteristics of the patients are presented in Table 1 and Table S1. Mean age of patients in the RG and LG groups was 60.0 ± 16.1 and 62.2 ± 12.7 years, respectively (MD = −0.91; 95% CI: −1.79 to 0.02; *p* = 0.04). Men accounted for 66.5% of the RG group compared to the LG group where the percentage of men was 61.9% (OR = 1.16; 95% CI: 1.05 to 1.28; *p* = 0.003). American Society of Anesthesiologists Physicial Status Classification ≥ III grade was concerned 16.9% of patients in RG group and 21.3% in LG group (OR = 0.86; 95%CI: 0.52 to 1.41; *p* = 0.55). In the RG group, neoadjuvant therapy was used statistically significantly more often than in the LG group (48.9% vs. 38.0%, respectively; OR = 1.67; 95%CI: 1.34 to 2.09; *p* < 0.001). Tumor distance from AV in the robotic (RG) and laparoscopic (LG) groups varied and amounted to 7.4 ± 3.5 vs. 8.5 ± 3.4 cm, respectively (MD = −0.72; 95% CI: −1.17 to −0.26; *p* < 0.001). A polled analysis of patients’ characteristics is presented in Table 2.

### 3.3. Intraoperative Period Characteristics

Detailed characteristics of the data concerning the intraoperative period are presented in Table 3. Pooled analysis showed that Hartman surgery was performed statistically significantly less frequently in the RG group compared to LG (3.8% vs. 5.2%, respectively; OR = 0.55; 95% CI: 0.31 to 0.98; *p* = 0.04). The inverse relationship was observed for the intersphincteric resection (19.4% vs. 13.4%; OR = 1.61; 95%CI: 1.10 to 2.35; *p* = 0.01).

Thirty-four articles reported the duration of surgery. The polled analysis showed that operative time in the RG group was 297.4 ± 99.3 min compared to 339.5 ± 359.2 min in the LG group (MD = 43.49; 95%CI: 25.26 to 61.51; *p* < 0.001; Figure S5). Operations using RG in comparison with LG were also associated with a statistically significantly lower frequency of conversion of the procedure to open surgery (2.6% vs. 7.3%; OR = 0.35; 95% CI: 0.26 to 0.46; *p* < 0.001; Figures S6 and S7). Intraoperative blood loss assessed from 24 studies was 224 ± 327.6 for robotic and 210.7 ± 305.2 mL for laparoscopic surgery (OR= −0.94; 95% CI: −30.11 to 28.22, *p* < 0.001; Figure S8), with blood transfusion required in 3.7% cases and 2.1%, respectively.

### 3.4. Pathological Evaluation

A polled analysis of pathological evaluation is presented in Table 4. There were no statistically significant differences between RG and LG groups in terms of TNM stage, N stage or lymph nodes harvested (Figure S9; *p* > 0.05). Circumferential margin (CRM) was positive in 4.1% (97/2338) in robotic and 4.4% (159/3616) in laparoscopic group (OR = 0.88; 95% CI: 0.67 to 1.16, *p* = 0.5). In the RG group, statistically significantly smaller tumor sizes were observed than in the LG group (3.4 ± 1.9 vs. 3.7 ± 2.2 cm, respectively; MD = −0.24; 95%CI: −0.42 to 0.07; *p* = 0.006), as well as the distal resection of margin was shorter than in the LG group (2.7 ± 1.9 vs. 2.9 ± 2.3cm; Md = −0.22; 95%CI: −0.32 to −0.11; *p* < 0.001).

### 3.5. Outcomes Evaluation

Survival to hospital discharge or 30-day overall survival rate was reported in 19 trials and was 99.6% for RG and 98.8% for LG (OR = 2.10; 95% CI: 1.00 to 4.43; *p* = 0.05; Figure S10). However, the pooled analysis did not show any advantage of any of the methods (RG or LG) in terms of OAS for longer periods of time (Table 5).). In addition, an analysis was carried out in subgroups depending on the region of the study, which showed that the equals in SHD between RG and LG groups were respectively: for Europe 98.6% vs. 97.9% (OR = 1.43; 95%CI: 0.64 to 3.18; *p* = 0.38), for Asia 99.9% vs. 98.9% (OR = 4.28; 95%CI: 0.95 to 19.16; *p* = 0.06), and for North America 98.2% vs. 98.0% (OR = 1.6; 95%CI: 0.49 to 5.28; *p* = 0.44).

The disease-free survival rate indicated a slight advantage of the robotic-assisted technique over the standard laparoscopic technique in all follow-up periods; however, these differences did not prove statistically significant.

Time to first flatus in the RG group was 2.5 ± 1.4 days and was statistically significantly shorter than in LG group (2.9 ± 2.0 days; MD = −0.34; 95% CI: −0.65 to 0.03; *p* = 0.03). In the case of time to a liquid diet, solid diet and bowel movement, the analysis showed no statistically significant differences (*p* > 0.05).

Length of hospital stay was reported in 34 studies. The polled analysis showed that the mean duration of hospital stay in the RG vs. LG group varied and amounted to 8.0 ± 5.3 vs. 9.5 ± 10.0 days (MD = −2.01; 95%CI: −2.90 to −1.11; *p* < 0.001; Figure S11).

Surgery with robotic-assisted technique compared to standard laparoscopic technique was associated with a statistically significantly lower risk of the urinary retention (3.5% vs. 6.1%, respectively; OR = 0.56; 95% CI: 0.34 to 0.92; *p* = 0.02; Figure S12). However, in the case of bowel obstruction, an inverse relationship was observed between RG and LG (5.1% vs. 2.7%; OR = 1.78; 95% CI: 1.05 to 3.03; *p* = 0.03; Figure S13). In the case of the remaining adverse events listed in Table 5, no statistically significant differences between the assessed surgical techniques were observed. Anastomotic leakage risk was comparable, estimated as 5.2% for robotic vs. 5.1% for laparoscopic surgery (OR 0.84; 95% CI: 0.65 to 1.07; *p* = 0.16; Figure S14).

## 4. Discussion

In our meta-analysis, we obtained new data based on the most recent literature. The survival to hospital discharge or 30-day overall survival rate was better in the RG than in the LG group (99.7% vs. 99.0%). Time to first flatus in the RG group was 2.5 ± 1.4 days and was statistically significantly shorter than in LG group (2.9 ± 2.0 days). We have also analyzed time to a liquid diet, solid diet and bowel movement, but no statistically significant differences were detected. Length of hospital stay in the RG was shorter compared to LG group (8.0 ± 5.3 vs. 9.5 ± 10.0 days).

Robot-assisted surgery of rectal cancer patients has been believed to overcome some of the difficulties present during the laparoscopic approach and thus improve its effectiveness. Better binocular vision with a three-dimensional view, wristed tools with better manoeuvrability, lack of tremor were enabling more exact operating in narrow pelvic space should improve the quality of specimen (intact mesorectal fascia and thus higher radicalness) and ensure saving anatomic structures essential to avoid adverse events. All those improvements should potentially lead to achieving the two most important goals in treating patients with cancer: better overall survival and higher quality of life.

Although some of the aspects analyzed in different studies show the robotic approach as superior to the laparoscopic one, overall survival has not been changed in favour of robotic-assisted surgery of the rectum. Our meta-analysis shown equivalent 5-years survival (85.6% for robotic and 87.6% for laparoscopic approach, *p* = 0.89). Interestingly, some data concerning pathologic aspects of the specimen, favouring individual methods (i.e., circumferential margin broader following robotic surgery 9.8 ± 7.1 vs. 8.8 ± 7.6, *p* = 0.42, but the almost equal ratio of positive CRM (4.1% and 4.4% respectively, *p* = 0.5). Thus R-TME is considered oncologically safe, as well as L-TME and open surgery, and different studies support the evidence [5,52,53,54,55,56].

These findings are contrary to the number of harvested lymph nodes, higher at laparoscopic approach (25.1 ± 25.2 vs. 20.5 ± 12.2, *p* < 0.001 with positive lymph nodes 7.3 ± 6.1 after laparoscopic resection and 2.5 ± 3.4 after robotic one, *p* < 0.001). Since the quality of surgical specimen can predict prognosis [57], all the differences should alter survival. However, all aspects mentioned above did not impact OS.

Although survival is shown to be the same at patients operated with analyzed tools, other essential advantages of robot-assisted surgery are shown in the meta-analysis, like lower urinary retention, lower urinary infection or ileus. Hospital stay was shorter in the R-TME group.

Some other benefits were assessed in some, not numerous studies, concerning the quality of life. Precise operating with better visibility gained with a three-dimensional view and wristed tools allow meticulous and sharp preparation of the hypogastric nerves and splanchnic plexus. This aspect of rectal surgery has been already improved by implementing Total Mesorectal Excision [1], in contrast to former blunt resection, in a study published by Kim et al. [38]. Although being a vast element of activity, genitourinary functions after R-TME has not been widely analyzed. Comparison of sexual functions according to international prostate symptom score (IPSS) showed the difference after three months (*p* = 0.036) following surgery favourable for R-TME (stronger sexual desire and better erectile functions) and equalization after six months. There was also earlier recovery concerning bladder functions after three months in the R-TME group, with stable voiding volume even right after the operation. The authors indicate adequate counter traction strength obtained through enhanced dexterity as an explanation of the results.

Enter et al. described the effects of TME performed at patients with low rectal tumours, operated using abdominoperineal resection (APR) describe the ratio of patients who maintained sexual functions as 57% compared to those operated with sphincter preservation (control group, 85%) [58]. They also indicate the OS worse than in the control group (60% vs. 81%). It is worth noting that a proven-value tool provides better circumferential margin and significantly better OS, equal to survival following anterior resection: ASAR (abdominal-sacral amputation of the rectum), described by Bebenek et al. [59]. Shiomi et al. showed the advantages of R-TME in especially challenging cases of lower rectal cancer in patients with visceral obesity. The complication rate, blood loss and hospital stay in patients with visceral obesity were significantly lower after R-TME than the laparoscopic approach. Operation time and pathologic results were similar in both groups, despite of high volume of visceral fat distorting surgical excision planes and leading to different complications [47]. The results of the meta-analysis show robotic-assisted rectal surgery as equally effective to the laparoscopic approach. Although more advanced, robotic-assisted rectal surgery does not influence overall survival. However, there are some benefits to using a higher quality of life, lower rates of sexual malfunctions in the period close to operation and better performance in, especially challenging situations.

### Limitations

Presented meta-analysis encompassed different types of publications, like randomized trials with patients matched according to different variables and single surgeons experience. Randomization on a 1:1 basis was strictly kept in randomized parallel-group trials like ROLARR [35] but was absent in single centre-experience, where the rates of preoperative chemoradiation were 43.2 vs. 19.5 [13]. Moreover, some more difficult cases were treated with robotic surgery due to subjective feeling of the outcome improvement following such approach shared by the authors. This could concern lower rectal localizations and more advanced stages demanding neoadjuvant treatment. Preoperative chemoradiation allows tumour downstaging and thus enables facilitated surgery. On the other hand, neoadjuvant treatment may also lead to oedema and fibrotic changes of irradiated tissues, making preparation more difficult and increasing smoke development and emission of fluid during surgery [13]. Some studies show more favourable outcomes following a robotic surgery, superior to laparoscopic at patients with unfavourable characteristics, i.e., neoadjuvant chemoradiation [29,60]. All those factors may influence the choice of procedure, either robotic or laparoscopic, across analyzed studies. This may lead to the different statistical distribution of patients using neoadjuvant treatment.

The compared techniques (RG and LG), apart from the differences in intraoperative parameters and outcomes demonstrated in the meta-analysis, also differ in their costs. The higher cost of the procedure in the case of RG and the cost of the device itself influence the lower availability of robotic-assisted surgery. As indicated by Siulva-Velazzco total cost of hospitalization of patients with RG is 15% higher than in patients operated with standard laparoscopic technique [48]. Ramji et al. [45] also indicate a significant increase in the cost of surgery with RG compared to LG, both in terms of operative room (123% increase in cost) and total cost per episode (59% increase in cost).

Robotic surgery has advantages in terms of the ergonomic design and expectations of shortening the learning curve, which may reduce the number of patients with adverse outcomes during a surgeon’s learning period [61]. Moreover, Jiménez-Rodríguez indicate that robotic advantages could have an impact on the learning curve for rectal cancer and lower the number of cases that are necessary for rectal resections [62]. Jiménez-Rodríguez et al. in another study shows that learning curve for robotic-assisted rectal cancer surgery is achieved after 21–23 cases [63] while as many studies indicate, a surgeon may become experienced in laparoscopic-assisted rectal surgery by operating 16–20 patients with rectal cancer [64,65].

## 5. Conclusions

Robotic-assisted techniques provide several advantages over laparoscopic techniques in reducing operative time and significantly lower conversion of the procedure to open surgery and a shorter duration of hospital stay and risk of urinary risk retention, urinary tract infection or ileus improving survival to hospital discharge or 30-day overall survival rate.

## Figures and Tables

**Table 1:** Patient characteristics.

Study	Country	Study Design	Robotic-Assisted Group			Laparoscopic Group		
			No.	Age	Sex, Male	No.	Age	Sex, Male
Ahmed et al. 2017	Portugal	PCD	99	69 ± 2	71.7%	85	68 ± 2	68.2%
Aselmann et al. 2018	Germany	R-PCD	44	61.1 ± 11.5	59.1%	41	65.1 ± 12.0	58.5%
Asklid et al. 2018	Sweden	RCS	72	65.4 ± 10.4	59.7%	47	70.1 ± 12.0	44.7%
Baek et al. 2010	Korea	PCD	41	65.6 ± 11.3	61.0%	41	64.4 ± 13.3	61.0%
Baek et al. 2012	Korea	PCD	154	59.1 ± 12.2	68.2%	150	62.3 ± 10.9	72.7%
Baek et al. 2013	Korea	RS	47	50.8 ± 12.9	66.0%	37	61.8 ± 12.8	75.7%
Baik et al. 2008	Korea	RCT	18	57.3 ± 6.3	77.8%	18	62.0 ± 9.0	77.8%
Barnajian et al. 2014	USA	RS	20	62.5 ± 11	60.0%	20	61.3 ± 13	60.0%
Bedirli et al. 2015	Turkey	RS	35	64.7 ± 8.5	68.6%	28	60.4 ± 7.1	67.9%
Bedrikovetski et al. 2020	Thailand	RS	117	61 ± 9.3	63.2%	1269	62.5 ± 13.7	57.9%
Bianchi et al. 2010	Italy	PCD	25	63.5 ± 9.3	72.0%	25	62.5 ± 13.7	68.0%
Bilgin et al. 2020	USA	R-PCD	72	59.0 ± 11.1	58.3%	44	57.2 ± 13.3	75.0%
Chen et al. 2017	Taiwan	RS	4744	NS	NS	5578	NS	NS
Cho et al. 2015	Korea	PCD	278	57.4 ± 11.6	65.5%	278	58.3 ± 10.4	66.2%
Corrigan et al. 2018	International	RCT	237	NS	67.9%	234	NS	67.9%
Crolla et al. 2018	Netherland	RS	168	67.0 ± 9.64	67.3%	184	68.1 ± 10.7	56.0%
D’Annibale et al. 2013	Italy	RS	50	66.0 ± 12.1	60.0%	50	65.7 ± 11.6	60.0%
de Jesus et al. 2016	Brazil	PCD	59	56.8 ± 14.7	61.0%	41	55.5 ± 16.7	58.0%
de’Angelis et al. 2020	France	PCD	50	64.4 ± 14.7	66.0%	81	55.5 ± 16.7	60.5%
Esen et al. 2018	Turkey	PCD	100	59 ± 11	60.0%	78	56 ± 13	65.0%
Feroci et al. 2016	Italy	RS	53	64.5 ± 12.1	50.9%	58	61.3 ± 13.6	72.4%
Garfinkle et al. 2019	Canada	RS	154	61.9 ± 13.5	68.8%	213	63.8 ± 13.3	59.6%
Ishihara et al. 2018	Japan	PCD	130	61.3	58.0%	234	64.1	65.0%
Jayne et al. 2017	UK	RCT	237	64.4 ± 11.0	67.9%	234	65.5 ± 11.9	67.9%
Kang 2013	Korea	PCD	165	61.2 ± 11.4	63.0%	165	60.4 ± 11.8	58.8%
Kethman 2020	USA	Cohrot	192	61.7	69.0%	206	62	63.4%
Kim 2012	Korea	PCD	30	54.13 ± 8.52	60.0%	39	56.85 ± 11.14	51.3%
Kim 2016	Korea	PCD	33	57.0 ± 9.6	69.7%	66	58.2 ± 9.8	69.7%
Kim 2018	Korea	RCT	66	60.4 ± 9.7	77.3%	73	59.7 ± 11.7	71.2%
Law 2016	China	PCD	220	63.5 ± 9.3	67.3%	171	63.3 ± 12.2	56.7%
Lim 2016	Korea	RS	74	65.1 ± 12.4	67.6%	64	65.8 ± 11.1	71.9%
Liu 2019	China	RS	80	62 ± 9.64	66.3%	116	59.57 ± 10.3	62.1%
Park 2011	Korea	PCD	52	57.3 ± 12.3	53.8%	123	65.1 ± 10.3	56.9%
Patriti 2009	Italy	PCD	29	68 ± 10	57.7%	37	69 ± 10	33.3%
Ramji 2016	Canada	RS	26	62.1 ± 9.1	73%	27	63.7 ± 11.2	70.0%
Rouanet 2018	France	RS	200	59.5 ± 10	65.5%	200	62 ± 8.5	68.0%
Shiomi 2016	Japan	RS	127	62 ± 9.3	73.2%	109	654 ± 10	59.6%
Silva-Velazco 2017	USA	RS	66	56 ± 13.8	75.8%	118	59.8 ± 9.8	55.9%
Sugoor 2018	India	PCD	100	48.7 ± 15.3	76.0%	113	49.2 ± 14.6	61.1%
Valverde 2017	France	PCD	65	67 ± 11	65.0%	65	65 ± 10	69.0%
Yamaguchi 2016	Japan	RS	203	64.8 ± 10.8	69.0%	239	65.9 ± 10.8	64.4%

Legend: NS = not specified; PCS = prospectively collected data; R-PCD = a retrospective analysis of prospectively collected data; RS = retrospective study.

**Table 2:** Polled analysis of patient characteristics among included trials.

Outcome	No. of Studies	Events/Participants or Mean ± SD		Events		Heterogeneity between Trials		*p*-Value for Differences across Groups
		Robotic	Laparoscopic	OR or MD	95%CI	*p*-Value	I^2^ Statistic	
Sex, male	39	2564/3858 (66.5%)	3345/5408 (61.9%)	1.16	1.05 to 1.28	0.32	9%	0.003
Age	37	60.0 ± 16.1	62.2 ± 12.7	−0.91	−1.79 to 0.02	<0.001	70%	0.04
BMI	31	25.1 ± 4.9	24.6 ± 4.3	0.14	−0.22 to 0.49	<0.001	78%	0.45
ASA score								
1 class	27	603/2358 (25.6%)	884/3628 (24.4%)	0.97	0.84 to 1.12	0.45	0%	0.70
2 class	27	1333/2358 (56.5%)	1878/3628 (51.8%)	1.19	0.99 to 1.44	0.001	51%	0.07
3 class	27	385/2358 (16.3%)	735/3628 (20.3%)	0.84	0.68 to 1.04	0.08	30%	0.11
4 class	27	13/2358 (1.6%)	36/3628 (3.5%)	0.90	0.44 to 1.83	0.83	0%	0.77
Neoadjuvant therapy	21	1000/2046 (48.9%)	1347/3541 (38.0%)	1.67	1.34 to 2.09	0.001	55%	<0.001
Tumour distance from AV (cm)	18	7.4 ± 3.5	8.5 ± 3.4	−0.72	−1.17 to −0.26	<0.001	79%	0.002
Tumour location								
Upper rectum	10	203/1385 (14.7%)	476/2867 (16.6%)	0.61	0.44 to 0.83	0.02	56%	0.002
Middle	10	631/1385 (45.6%)	1254/2867 (43.7%)	1.11	0.93 to 1.32	0.21	25%	0.24
Lower	14	800/1919 (41.7%)	1334/3457 (38.6%)	1.18	0.92 to 1.52	<0.001	71%	0.18

Legend: BMI = Body Mass index; CI: confidence interval; MD = mean difference; OR = odds ratio; SD = standard deviation. Note: Not all outcomes were reported in every study. “No. of studies” refers to the studies included in the analysis for the particular outcome.

**Table 3:** Polled analysis of intraoperative parameters among included trials.

Outcome	No. of Studies	Events/Participants or Mean ± SD		Events		Heterogeneity between Trials		*p*-Value for Differences across Groups
		Robotic	Laparoscopic	OR or MD	95%CI	*p*-Value	I^2^ Statistic	
Surgical procedure								
Full TME	4	124/285 (43.5%)	99/246 (40.2%)	1.37	0.36 to 5.26	<0.001	84%	0.64
LAR	21	1894/2569 (73.7%)	1996/2868 (69.6%)	1.11	0.86 to 1.44	<0.001	71%	0.42
APR	15	252/1546 (16.3%)	303/1558 (19.4%)	0.88	0.62 to 1.25	0.01	51%	0.47
Hartman	10	47/1241 (3.8%)	76/1449 (5.2%)	0.55	0.31 to 0.98	0.84	0%	0.04
ISR	5	118/608 (19.4%)	109/814 (13.4%)	1.61	1.10 to 2.35	0.20	33%	0.01
Operative time (min)	34	297.4 ± 99.3	339.5 ± 359.2	43.39	25.26 to 61.51	<0.001	98%	<0.001
Diverting ileostomy	16	1102/1831 (60.2%)	1016/1815 (56.0%)	1.07	0.87 to 1.31	0.12	31%	0.53
Intraoperative blood loss (mL)	24	224 ± 327.6	210.7 ± 305.2	−0.94	−30.11 to 28.22	<0.001	98%	0.95
Conversion to open	30	76/2917 (2.6%)	236/3255 (7.3%)	0.35	0.26 to 0.46	0.56	0%	<0.001
Intraoperative complications	4	45/445 (10.1%)	108/1599 (6.8%)	−0.00	−0.03 to 0.02	0.64	0%	0.76
Haemorrhage	3	7/327 (2.1%)	12/328 (3.7%)	−0.01	−0.05 to 0.03	0.16	46%	0.78
Transfusion	3	6/162 (3.7%)	6/285 (2.1%)	0.02	−0.01 to 0.05	0.95	0%	0.32

Legend: APR = abdominoperineal resection; CI: confidence interval; ISR = intersphincteric resection; LAR = low anterior resection; MD = mean difference; OR = odds ratio; SD = standard deviation; TME = total mesorectal excision. Note: Not all outcomes were reported in every study. “No. of studies” refers to the studies included in the analysis for the particular outcome.

**Table 4:** Polled analysis of pathological evaluation.

Outcome	No. of Studies	Events/Participants or Mean ± SD		Events		Heterogeneity between Trials		*p*-Value for Differences across Groups
		Robotic	Laparoscopic	OR or MD	95%CI	*p*-Value	I^2^ Statistic	
**TNM stage**								
Stage I	13	221/1169 (18.9%)	265/2397 (11.1%)	0.94	0.75 to 1.17	0.84	0%	0.58
Stage II	11	237/986 (24.0%)	226/1010 (22.4%)	1.13	0.91 to 1.41	0.75	0%	0.26
Stage III	11	410/986 (41.6%)	458/1010 (45.3%)	0.86	0.71 to 1.04	0.88	0%	0.12
Stage IV	3	6/117 (5.1%)	6/125 (4.8%)	1.11	0.31 to 3.94	0.38	0%	0.87
N stage								
N0	16	1150/1829 (62.9%)	1252/3024 (41.4%)	1.05	0.88 to 1.25	0.15	28%	0.58
N1	15	423/1803 (23.5%)	669/2997 (22.3%)	1.04	0.86 to 1.25	0.17	26%	0.67
N2	12	139/1494 (9.3%)	247/2741 (9.0%)	0.96	0.76 to 1.22	0.92	0%	0.75
Lymph nodes harvested	34	20.5 ± 12.2	25.1 ± 25.2	−0.05	−1.06 to 0.96	<0.001	85%	0.92
Positive lymph nodes	4	2.5 ± 3.4	7.3 ± 6.1	−1.42	−4.53 to 1.69	<0.001	98%	0.37
Tumour size (cm)	11	3.4 ± 1.9	3.7 ± 2.2	−0.24	−0.42 to −0.07	0.37	7%	0.006
CRM (mm)	7	9.8 ± 7.1	8.8 ± 7.6	0.08	−1.03 to 1.19	0.42	0%	0.88
CRM positive	22	97/2338 (4.1%)	159/3616 (4.4%)	0.88	0.67 to 1.16	0.50	0%	0.36
DRM (cm)	20	2.7 ± 1.9	2.9 ± 2.3	−0.22	−0.32 to −0.11	<0.001	87%	<0.001
DRM positive	4	3/286 (1.0%)	3/343 (0.9%)	0.97	0.21 to 4.46	0.44	0%	0.96
PRM (cm)	7	12.6 ± 6.2	13.0 ± 6.6	0.30	−0.25 to 0.86	0.008	66%	0.28
Lymphovascular invasion	4	112/567 (19.8%)	101/588 (17.2%)	1.27	0.94 to 1.72	0.50	0%	0.12

Legend: CI: confidence interval; CRM = Circumferential resection margin; DRM = Distal resection margin; MD = mean difference; OR = odds ratio; PRM = proximal resection margin; SD = standard deviation. Note: Not all outcomes were reported in every study. “No. of studies” refers to the studies included in the analysis for the particular outcome.

**Table 5:** Polled analysis of outcomes among included trials.

Outcome	No. of Studies	Events/Participants or Mean ± SD		Events		Heterogeneity between Trials		*p*-Value for Differences across Groups
		Robotic	Laparoscopic	OR or MD	95%CI	*p*-Value	I^2^ Statistic	
**Overall survival, OAS**								
SHD/30—days	18	6346/6369 (99.6%)	8219/8319 (98.8%)	2.10	1.00 to 4.43	0.07	43%	0.05
1—yr	1	44/44 (100%)	37/41 (90.3%)	10.68	0.56 to 204.84	NA	NA	0.12
3—yrs	4	320/371 (86.3%)	316/363 (87.1%)	1.02	0.56 to 1.83	0.21	34%	0.96
5—yrs	4	551/644 (85.6%)	488/557 (87.6%)	0.87	0.61 to 1.23	0.89	0%	0.43
**The disease—free survival rate, DFS**								
1—yr	1	41/44 (93.2%)	32/41 (78.0%)	3.84	0.96 to 15.37	NA	NA	0.06
2—yrs	3	131/171 (76.6%)	121/163 (74.2%)	1.15	0.65 to 2.04	0.29	20%	0.62
3—yrs	3	346/424 (81.6%)	307/386 (79.5%)	1.13	0.79 to 1.60	0.97	0%	0.51
5—yrs	1	138/154 (89.6%)	135/165 (81.8%)	1.14	0.64 to 2.01	NA	NA	0.66
Time to liquid diet (days)	7	3.7 ± 2.4	4.4 ± 2.6	−0.58	−1.50 to 0.33	<0.001	95%	0.95
Time to solid diet (days)	9	4.4 ± 3.0	5.1 ± 2.8	−0.46	−0.95 to 0.03	<0.001	75%	0.07
Time to flatus (days)	13	2.5 ± 1.4	2.9 ± 2.0	−0.34	−0.65 to −0.03	<0.001	85%	0.03 *
Time to bowel movement (days)	7	2.4 ± 1.9	2.4 ± 1.7	−0.06	−0.25 to 0.13	0.49	0%	0.53
Hospital length of stay (days)	34	8.0 ± 5.3	9.5 ± 10.0	−2.01	−2.90 to −1.11	<0.001	99%	<0.001 *
Readmission rate	11	91/882 (10.3%)	203/2066 (9.8%)	1.14	0.82 to 1.60	0.38	6%	0.44
Reoperation rate	13	67/1061 (6.3%)	80/1120 (7.1%)	0.87	0.61 to 1.25	0.47	0%	0.46
**Adverse events**								
Overall, 30—days complications	18	685/2520 (27.2%)	1453/7639 (19.0%)	1.11	0.80 to 1.55	<0.001	84%	0.53
*Anastomotic leakage*	28	135/2607 (5.2%)	208/4097 (5.1%)	0.84	0.65 to 1.07	0.66	0%	0.16
*Anastomotic bleeding*	4	10/405 (2.5%)	9/416 (2.2%)	1.17	0.46 to 2.98	0.91	0%	0.75
*Parastomal/trocar hernia*	3	45/1081 (4.2%)	189/4943 (3.8%)	1.23	0.88 to 1.73	0.65	0%	0.23
*Urinary retention*	12	51/1455 (3.5%)	96/1560 (6.1%)	0.56	0.34 to 0.92	0.13	33%	0.02 *
*Urinary tract infection*	6	12/673 (1.8%)	26/839 (3.1%)	0.62	0.30 to 1.27	0.74	0%	0.19
*Ureteral injury*	3	0/253 (0.0%)	4/243 (1.6%)	0.25	0.04 to 1.56	0.97	0%	0.14
Bowel obstruction	5	28/549 (5.1%)	48/1750 (2.7%)	1.78	1.05 to 3.03	0.55	0%	0.03 *
Small bowel perforation	5	4/826 (0.5%)	14/876 (1.6%)	0.39	0.14 to 1.11	0.77	0%	0.08
Wound infection	22	130/7048 (1.8%)	229/9299 (2.5%)	0.79	0.62 to 1.00	0.66	0%	0.05
Sepsis	4	10/381 (2.6%)	99/1644 (6.0%)	0.79	0.39 to 1.59	0.65	0%	0.51
Wound dehiscence	5	5/444 (1.1%)	35/1714 (2.0%)	1.04	0.25 to 4.42	0.28	22%	0.96
Abdominal bleeding	12	16/2052 (0.8%)	144/6015 (2.4%)	0.85	0.30 to 2.35	0.03	49%	0.75
Ileus	19	787/6363 (12.4%)	1221/8637 (14.1%)	0.94	0.77 to 1.14	0.32	11%	0.51
Abdominal abscess	8	14/911 (1.5%)	15/984 (1.5%)	0.90	0.43 to 1.91	0.84	0%	0.79
Pelvic abscess	3	10/251 (4.0%)	10/303 (3.3%)	1.20	0.40 to 3.65	0.32	12%	0.74
Fistula	5	22/784 (2.8%)	13/762 (1.7%)	1.66	0.83 to 3.33	0.73	0%	0.15
Pulmonary embolism	3	1/261 (0.4%)	2/372 (0.5%)	0.75	0.09 to 6.15	0.50	0%	0.79
Deep vein thrombosis	3	4/440 (0.9%)	3/502 (0.6%)	1.86	0.24 to 14.26	0.25	27%	0.55
Acute renal failure	5	1/351 (0.3%)	5/526 (1.0%)	0.62	0.15 to 2.57	0.82	0%	0.51
Acute renal failure	6	34/1223 (2.8%)	210/5263 (4.0%)	0.85	0.59 to 1.22	0.89	0%	0.38
Peripheral nerve injury	1	0/66 (0.0%)	0/118 (0.0%)	NE	NE	NA	NA	NA

Legend: CI: confidence interval; MD = mean difference; NA = not applicable; NE = not estimable; OR = odds ratio; SD = standard deviation. Note: Not all outcomes were reported in every study. “No. of studies” refers to the studies included in the analysis for the particular outcome. * *p* < 0.05 statistically significant.

## Supplementary Materials

The following are available online at https://www.mdpi.com/article/10.3390/cancers14010180/s1, Figure S1: A summary table of review authors’ judgements for each risk of bias item for each randomized study, Figure S2: A plot of the distribution of review authors’ judgements across randomized studies for each risk of bias item, Figure S3: A summary table of review authors’ judgements for each risk of bias item for each non-randomized study, Figure S4: A plot of the distribution of review authors’ judgements across non-randomized studies for each risk of bias item, Figure S5: Forest plot of operative time in the robotic-assisted and laparoscopic groups, Figure S6: Forest plot of conversion to open surgery rate in the robotic-assisted and laparoscopic groups, Figure S7: Funnel plot with 95% confidence limits for publication bias in the studies investigating: (A) conversion to open surgery, (B) survival to hospital discharge, (C) urinary retention occurrence, (D) anastomotic leakage occurrence, Figure S8: Forest plot of intraoperative blood loss in the robotic-assisted and laparoscopic groups, Figure S9: Forest plot of lymph nodes harvested in the robotic-assisted and laparoscopic groups, Figure S10: Forest plot of survival to hospital discharge in the robotic-assisted and laparoscopic groups, Figure S11: Forest plot of hospital length of stay in the robotic-assisted and laparoscopic groups, Figure S12: Forest plot of urinary retention rate in the robotic-assisted and laparoscopic groups, Figure S13: Forest plot of bowel obstruction rate in the robotic-assisted and laparoscopic groups, Figure S14: Forest plot of anastomotic leakage rate in the robotic-assisted and laparoscopic groups, Table S1: PRISMA checklist.

## References

[1] Heald R.J., Husband E.M., Ryall R.D.H. (1982). The mesorectum in rectal cancer surgery—The clue to pelvic recurrence?. Br. J. Surg..

[2] Van der Pas M.H., Haglind E., Cuesta M.A., Fürst A., Lacy A.M., Hop W.C., Bonjer H.J. (2013). COlorectal cancer Laparoscopic or Open Resection II (COLOR II) Study Group. Laparoscopic versus open surgery for rectal cancer (COLOR II): Short-term outcomes of a randomized, phase 3 trial. Lancet Oncol..

[3] Green B.L., Marshall H.C., Collinson F., Quirke P., Guillou P., Jayne D.G., Brown J.M. (2013). Long-term follow-up of the Medical Research Council CLASSIC trial of conventional versus laparoscopically assisted resection in colorectal cancer. Br. J. Surg..

[4] Kang S.B., Park J.W., Jeong S.Y., Nam B.H., Choi H.S., Kim D.W., Lim S.B., Lee T.G., Kim D.Y., Kim J.S. (2010). Open versus laparoscopic surgery for mid or low rectal cancer after neoadjuvant chemoradiotherapy (KOREAN trial): Short-term outcomes of an open-label randomized controlled trial. Lancet Oncol..

[5] Pigazzi A., Ellenhorn J.D.I., Ballantyne G.H., Paz I.B. (2006). Robotic-assisted laparoscopic low anterior resection with total mesorectal excision for rectal cancer. Surg. Endosc..

[6] Page M.J., McKenzie J.E., Bossuyt P.M., Boutron I., Hoffmann T.C., Mulrow C.D., Shamseer L., Tetzlaff J.M., Akl E.A., Brennan S.E. (2021). The PRISMA 2020 statement: An updated guideline for reporting systematic reviews. Syst. Rev..

[7] Sterne J.A.C., Savović J., Page M.J., Elbers R.G., Blencowe N.S., Boutron I., Cates C.J., Cheng H.-Y., Corbett M.S., Eldridge S.M. (2019). RoB 2: A revised tool for assessing risk of bias in randomised trials. BMJ.

[8] Sterne J.A.C., Hernán M.A., Reeves B.C., Savović J., Berkman N.D., Viswanathan M., Henry D., Altman D.G., Ansari M.T., Boutron I. (2016). ROBINS-I: A tool for assessing risk of bias in non-randomised studies of interventions. BMJ.

[9] McGuinness L.A., Higgins J.P.T. (2021). Risk-of-bias VISualization (robvis): An R package and Shiny web app for visualizing risk-of-bias assessments. Res. Synth. Methods.

[10] Hozo S.P., Djulbegovic B., Hozo I. (2005). Estimating the mean and variance from the median, range, and the size of a sample. BMC Med. Res. Methodol..

[11] Higgins J.P.T., Altman D.G., Gøtzsche P.C., Jüni P., Moher D., Oxman A.D., Savovic J., Schulz K.F., Weeks L., Sterne J.A.C. (2011). The Cochrane Collaboration’s tool for assessing risk of bias in randomised trials. BMJ.

[12] Ahmed J., Cao H., Panteleimonitis S., Khan J., Parvaiz A. (2017). Robotic vs. laparoscopic rectal surgery in high-risk patients. Color. Dis..

[13] Aselmann H., Kersebaum J., Bernsmeier A., Beckmann J.H., Möller T., Egberts J.H., Schafmayer C., Röcken C., Becker T. (2018). Robotic-assisted total mesorectal excision (TME) for rectal cancer results in a significantly higher quality of TME specimen compared to the laparoscopic approach—Report of a single-center experience. Int. J. Color. Dis..

[14] Asklid D., Gerjy R., Hjern F., Pekkari K., Gustafsson U.O. (2019). Robotic vs. laparoscopic rectal tumour surgery: A cohort study. Color. Dis..

[15] Baek J.-H., Pastor C., Pigazzi A. (2011). Robotic and laparoscopic total mesorectal excision for rectal cancer: A case-matched study. Surg. Endosc..

[16] Baek S.-J., Kim S.-H., Cho J.-S., Shin J.-W., Kim J. (2012). Robotic versus Conventional Laparoscopic Surgery for Rectal Cancer: A Cost Analysis from A Single Institute in Korea. World J. Surg..

[17] Baek S.J., Al-Asari S., Jeong D.H., Hur H., Min B.S., Baik S.H., Kim N.K. (2013). Robotic versus laparoscopic coloanal anastomosis with or without intersphincteric resection for rectal cancer. Surg. Endosc..

[18] Baik S.H., Ko Y.T., Kang C.M., Lee W.J., Kim N.K., Sohn S.K., Chi H.S., Cho C.H. (2008). Robotic tumor-specific mesorectal excision of rectal cancer: Short-term outcome of a pilot randomized trial. Surg. Endosc..

[19] Barnajian M., Pettet D., Kazi E., Foppa C., Bergamaschi R. (2014). Quality of total mesorectal excision and depth of circum-ferential resection margin in rectal cancer: A matched comparison of the first 20 robotic cases. Color. Dis..

[20] Bedirli A., Salman B., Yuksel O. (2016). Robotic Versus Laparoscopic Resection for Mid and Low Rectal Cancers. JSLS J. Soc. Laparoendosc. Surg..

[21] Bedrikovetski S., Dudi-Venkata N.N., Kroon H.M., Moore J.W., Hunter R.A., Sammour T. (2020). Outcomes of Minimally Invasive Versus Open Proctectomy for Rectal Cancer: A Propensity-Matched Analysis of Bi-National Colorectal Cancer Audit Data. Dis. Colon Rectum.

[22] Bianchi P.P., Ceriani C., Locatelli A., Spinoglio G., Zampino M.G., Sonzogni A., Crosta C., Andreoni B. (2010). Robotic versus laparoscopic total mesorectal excision for rectal cancer: A comparative analysis of oncological safety and short-term outcomes. Surg. Endosc..

[23] Bilgin I.A., Bas M., Aytac E., Benlice C., Esen E., Kirbiyik E., Kiziltas C., Aghayeva A., Ozben V., Hamzaoglu I. (2020). Operative and long-term oncological outcomes in patients undergoing robotic versus laparoscopic surgery for rectal cancer. Int. J. Med. Robot. Comput. Assist. Surg..

[24] Chen S.-T., Wu M.-C., Hsu T.-C., Yen D.W., Chang C.-N., Hsu W.-T., Wang C.-C., Lee M., Liu S.-H., Lee C.-C. (2018). Comparison of outcome and cost among open, laparoscopic, and robotic surgical treatments for rectal cancer: A propensity score matched analysis of nationwide inpatient sample data. J. Surg. Oncol..

[25] Cho M.S., Baek S.J., Hur H., Min B.S., Baik S.H., Lee K.Y., Kim N.K. (2015). Short and long-term outcomes of robotic versus laparoscopic total mesorectal excision for rectal cancer: A case-matched retrospective study. Medicine.

[26] Corrigan N., Marshall H., Croft J., Copeland J., Jayne D., Brown J. (2018). Exploring and adjusting for potential learning effects in ROLARR: A randomised controlled trial comparing robotic-assisted vs. standard laparoscopic surgery for rectal cancer resection. Trials.

[27] Crolla R., Mulder P.G., van der Schelling G.P. (2018). Does robotic rectal cancer surgery improve the results of experienced laparo-scopic surgeons? An observational single institution study comparing 168 robotic assisted with 184 laparoscopic rectal resections. Surg. Endosc..

[28] D’Annibale A., Pernazza G., Monsellato I., Pende V., Lucandri G., Mazzocchi P., Alfano G. (2013). Total mesorectal excision: A comparison of oncological and functional outcomes between robotic and laparoscopic surgery for rectal cancer. Surg. Endosc..

[29] De Jesus J.P., Valadão M., Araujo R.O.D.C., Cesar D., Linhares E., Iglesias A.C. (2016). The circumferential resection margins status: A comparison of robotic, laparoscopic and open total mesorectal excision for mid and low rectal cancer. Eur. J. Surg. Oncol..

[30] De’Angelis N., Notarnicola M., Martínez-Pérez A., Memeo R., Charpy C., Urciuoli I., Maroso F., Sommacale D., Amiot A., Canouï-Poitrine F. (2020). Robotic Versus Laparoscopic Partial Mesorectal Excision for Cancer of the High Rectum: A Single-Center Study with Propensity Score Matching Analysis. World J. Surg..

[31] Esen E., Aytac E., Ağcaoğlu O., Zenger S., Balik E., Baca B., Hamzaoğlu I., Karahasanoğlu T., Buğra D. (2018). Totally Robotic Versus Totally Laparoscopic Surgery for Rectal Cancer. Surg. Laparosc. Endosc. Percutaneous Tech..

[32] Feroci F., Vannucchi A., Bianchi P.P., Cantafio S., Garzi A., Formisano G., Scatizzi M. (2016). Total mesorectal excision for mid and low rectal cancer: Laparoscopic vs. robotic surgery. World J. Gastroenterol..

[33] Garfinkle R., Abou-Khalil M., Bhatnagar S., Wong-Chong N., Azoulay L., Morin N., Vasilevsky C.-A., Boutros M. (2019). A Comparison of Pathologic Outcomes of Open, Laparoscopic, and Robotic Resections for Rectal Cancer Using the ACS-NSQIP Proctectomy-Targeted Database: A Propensity Score Analysis. J. Gastrointest. Surg..

[34] Ishihara S., Kiyomatsu T., Kawai K., Tanaka T., Hata K., Kazama S., Sunami E., Nozawa H., Watanabe T. (2018). The short-term outcomes of robotic sphincter-preserving surgery for rectal cancer: Comparison with open and laparoscopic surgery using a propensity score analysis. Int. J. Color. Dis..

[35] Jayne D., Pigazzi A., Marshall H., Croft J., Corrigan N., Copeland J., Quirke P., West N., Rautio T., Thomassen N. (2017). Effect of Robotic-Assisted vs. Conventional Laparoscopic Surgery on Risk of Conversion to Open Laparotomy Among Patients Undergoing Resection for Rectal Cancer: The ROLARR Randomized Clinical Trial. JAMA.

[36] Kang J., Yoon K.J., Min B.S., Hur H., Baik S.H., Kim N.K., Lee K.Y. (2013). The impact of robotic surgery for mid and low rectal cancer: A case-matched analysis of a 3-arm comparison-open, laparoscopic, and robotic surgery. Ann. Surg..

[37] Kethman W.C., Harris A.H., Morris A.M., Shelton A., Kirilcuk N., Kin C. (2020). Oncologic and Perioperative Outcomes of Laparoscopic, Open, and Robotic Approaches for Rectal Cancer Resection: A Multicenter, Propensity Score-Weighted Cohort Study. Dis. Colon Rectum.

[38] Kim J.Y., Kim N.-K., Lee K.Y., Hur H., Min B.S., Kim J.H. (2012). A Comparative Study of Voiding and Sexual Function after Total Mesorectal Excision with Autonomic Nerve Preservation for Rectal Cancer: Laparoscopic Versus Robotic Surgery. Ann. Surg. Oncol..

[39] Kim Y.S., Kim M.J., Park S.C., Sohn D.K., Kim D.Y., Chang H.J., Nam B.-H., Oh J.H. (2016). Robotic Versus Laparoscopic Surgery for Rectal Cancer after Preoperative Chemoradiotherapy: Case-Matched Study of Short-Term Outcomes. Cancer Res. Treat..

[40] Kim M.J., Park S.C., Park J.W., Chang H.J., Kim D.Y., Nam B.H., Sohn D.K., Oh J.H. (2018). Robot-assisted Versus Laparoscopic Surgery for Rectal Cancer: A Phase II Open Label Prospective Randomized Controlled Trial. Ann. Surg..

[41] Lim D.R., Bae S.U., Hur H., Min B.S., Baik S.H., Lee K.Y., Kim N.K. (2017). Long-term oncological outcomes of robotic versus laparoscopic total mesorectal excision of mid–low rectal cancer following neoadjuvant chemoradiation therapy. Surg. Endosc..

[42] Liu W.-H., Yan P., Hu D.-P., Jin P.-H., Lv Y.-C., Liu R., Yang X.-F., Yang K.-H., Guo T.-K. (2019). Short-Term Outcomes of Robotic versus Laparoscopic Total Mesorectal Excision for Rectal Cancer: A Cohort Study. Am. Surg..

[43] Park J.S., Choi G.-S., Lim K.H., Jang Y.S., Jun S.H. (2011). S052: A comparison of robot-assisted, laparoscopic, and open surgery in the treatment of rectal cancer. Surg. Endosc..

[44] Patriti A., Ceccarelli G., Bartoli A., Spaziani A., Biancafarina A., Casciola L. (2009). Short- and medium-term outcome of robot-assisted and traditional laparoscopic rectal resection. JSLS.

[45] Ramji K.M., Cleghorn M.C., Josse J.M., MacNeill A., O’Brien C., Urbach D., Quereshy F.A. (2016). Comparison of clinical and economic outcomes between robotic, laparoscopic, and open rectal cancer surgery: Early experience at a tertiary care center. Surg. Endosc..

[46] Rouanet P., Bertrand M.M., Jarlier M., Mourregot A., Traore D., Taoum C., De Forges H., Colombo P.-E. (2018). Robotic Versus Laparoscopic Total Mesorectal Excision for Sphincter-Saving Surgery: Results of a Single-Center Series of 400 Consecutive Patients and Perspectives. Ann. Surg. Oncol..

[47] Shiomi A., Kinugasa Y., Yamaguchi T., Kagawa H., Yamakawa Y. (2016). Robot-assisted versus laparoscopic surgery for lower rectal cancer: The impact of visceral obesity on surgical outcomes. Int. J. Color. Dis..

[48] Silva-Velazco J., Dietz D.W., Stocchi L., Costedio M., Gorgun E., Kalady M.F., Kessler H., Lavery I.C., Remzi F.H. (2017). Considering Value in Rectal Cancer Surgery: An Analysis of Costs and Outcomes Based on the Open, Laparoscopic, and Robotic Approach for Proctectomy. Ann. Surg..

[49] Sugoor P., Verma K., Chaturvedi A., Kannan S., Desouza A., Ostwal V., Engineer R., Saklani A. (2019). Robotic versus laparoscopic sphincter-preserving total mesorectal excision: A propensity case-matched analysis. Int. J. Med. Robot. Comput. Assist. Surg..

[50] Valverde A., Goasguen N., Oberlin O., Svrcek M., Fléjou J.-F., Sezeur A., Mosnier H., Houdart R., Lupinacci R.M. (2017). Robotic versus laparoscopic rectal resection for sphincter-saving surgery: Pathological and short-term outcomes in a single-center analysis of 130 consecutive patients. Surg. Endosc..

[51] Yamaguchi T., Kinugasa Y., Shiomi A., Tomioka H., Kagawa H., Yamakawa Y. (2016). Robotic-assisted vs. conventional laparoscopic surgery for rectal cancer: Short-term outcomes at a single center. Surg. Today.

[52] Lanfranco A.R., Castellanos A.E., Desai J.P., Meyers W.C. (2004). Robotic surgery: A current perspective. Ann. Surg..

[53] D’Annibale A., Morpurgo E., Fiscon V., Trevisan P., Sovernigo G., Orsini C., Guidolin D. (2004). Robotic and Laparoscopic Surgery for Treatment of Colorectal Diseases. Dis. Colon Rectum.

[54] Hellan M., Anderson C., Ellenhorn J.D.I., Paz B., Pigazzi A. (2007). Short-Term Outcomes After Robotic-Assisted Total Mesorectal Excision for Rectal Cancer. Ann. Surg. Oncol..

[55] Spinoglio G., Summa M., Priora F., Quarati R., Testa S. (2007). Robotic Colorectal Surgery: First 50 Cases Experience. Dis. Colon Rectum.

[56] Baik S.H., Lee W.J., Rha K.H., Kim N.-K., Sohn S.K., Chi H.S., Cho C.H., Kil Lee S., Cheon J.H., Ahn J.B. (2007). Robotic total mesorectal excision for rectal cancer using four robotic arms. Surg. Endosc..

[57] Nagtegaal I.D., Van De Velde C.J., Van Der Worp E., Kapiteijn E., Quirke P., Van Krieken J.H.J. (2002). the Pathology Review Committee for the Cooperative Clinical Investigators of the Dutch Colorectal Cancer Group Macroscopic Evaluation of Rectal Cancer Resection Specimen: Clinical Significance of the Pathologist in Quality Control. J. Clin. Oncol..

[58] Enker W.E., Havenga K., Polyak T., Thaler H., Cranor M. (1997). Abdominoperineal Resection via Total Mesorectal Excision and Autonomic Nerve Preservation for Low Rectal Cancer. World J. Surg..

[59] Bebenek M., Pudełko M., Cisarz K., Balcerzak A., Tupikowski W., Wojciechowski L., Stankowska A., Tarkowski R., Szulc R. (2007). Therapeutic results in low-rectal cancer patients treated with abdominosacral resection are similar to those obtained using anterior resection in mid-and upper-rectal cancer cases. Eur. J. Surg. Oncol..

[60] Staderini F., Foppa C., Minuzzo A., Badii B., Qirici E., Trallori G., Mallardi B., Lami G., Macrì G., Bonanomi A. (2016). Robotic rectal surgery: State of the art. World J. Gastrointest. Oncol..

[61] Noh G.T., Han M., Hur H., Baik S.H., Lee K.Y., Kim N.K., Min B.S. (2021). Impact of laparoscopic surgical experience on the learning curve of robotic rectal cancer surgery. Surg. Endosc..

[62] Jiménez-Rodríguez R.M., Rubio-Dorado-Manzanares M., Díaz-Pavón J.M., Reyes-Díaz M.L., Vazquez-Monchul J.M., Garcia-Cabrera A.M., Padillo J., De la Portilla F. (2016). Learning curve in robotic rectal cancer surgery: Current state of affairs. Int. J. Color. Dis..

[63] Jiménez-Rodríguez R.M., Díaz-Pavón J.M., de la Portilla de Juan F., Prendes-Sillero E., Dussort H.C., Padillo J. (2013). Learning curve for robotic-assisted laparoscopic rectal cancer surgery. Int. J. Color. Dis..

[64] Liang J.-W., Zhang X.-M., Zhou Z.-X., Wang Z., Bi J.-J. (2011). Learning curve of laparoscopic-assisted surgery for rectal cancer. Zhonghua Yi Xue Za Zhi.

[65] Clinical Outcomes of Surgical Therapy Study Group (2004). A comparison of laparoscopically assisted and open colectomy for colon cancer. N. Engl. J. Med..

