# Supplementary material for: Robotic-Assisted vs. Standard Laparoscopic Surgery for Rectal Cancer Resection: A Systematic Review and Meta-Analysis of 19,731 Patients
Source: Cancers (Basel). 2021 Dec 30;14(1):180. doi: 10.3390/cancers14010180 (PMC8750860; doi:10.3390/cancers14010180)
Supplement: Supplementary file 1 [file cancers-14-00180-s001.zip › cancers-1518528-supplementary.pdf]

Robotic-Assisted vs. Standard Laparoscopic Surgery for Rectal Cancer Resection: A Systematic Review and Meta-Analysis of 19,731 Patients

Supplementary Digital File

CONTENT

Figure S1. A summary table of review authors' judgements for each risk of bias item for each randomized study. 2

Figure S2. A plot of the distribution of review authors' judgements across randomized studies for each risk of bias item. 2

Figure S3. A summary table of review authors' judgements for each risk of bias item for each non-randomized study. 3

Figure S4. A plot of the distribution of review authors' judgements across non-randomized studies for each risk of bias item. 4

Figure S5. Forest plot of operative time in the robotic-assisted and laparoscopic groups.. 4

Figure S6. Forest plot of conversion to open surgery rate in the robotic-assisted and laparoscopic groups. 5

Figure S7. Funnel plot with 95% confidence limits for publication bias in the studies investigating: (A) conversion to open surgery, (B) survival to hospital discharge, (C) urinary retention occurrence, (D) anastomotic leakage occurrence. 6

Figure S8. Forest plot of intraoperative blood loss in the robotic-assisted and laparoscopic groups. 6

Figure S9. Forest plot of lymph nodes harvested in the robotic-assisted and laparoscopic groups. . 7

Figure S10. Forest plot of survival to hospital discharge in the robotic-assisted and laparoscopic groups. 7

Figure S11. Forest plot of hospital length of stay in the robotic-assisted and laparoscopic groups.. 8

Figure S12. Forest plot of urinary retention rate in the robotic-assisted and laparoscopic groups... 8

Figure S13. Forest plot of bowel obstruction rate in the robotic-assisted and laparoscopic groups..9

Figure S14. Forest plot of anastomotic leakage rate in the robotic-assisted and laparoscopic groups.. 9

Table S1. PRISMA checklist 10

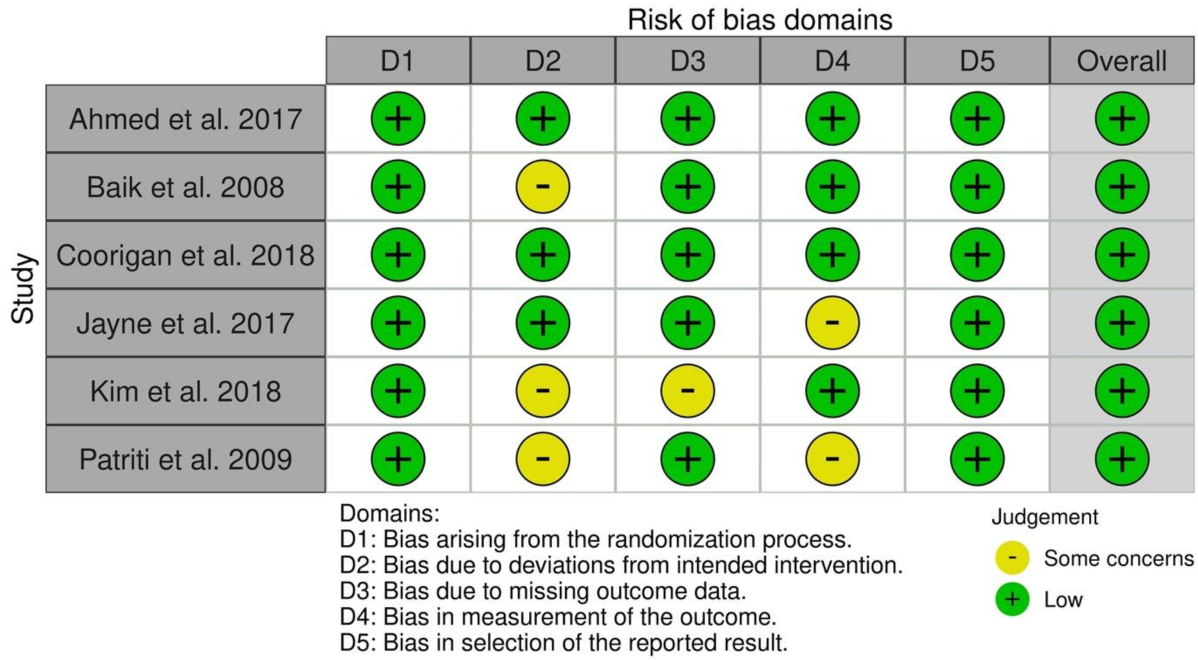

Figure S1. A summary table of review authors' judgements for each risk of bias item for each randomized study.

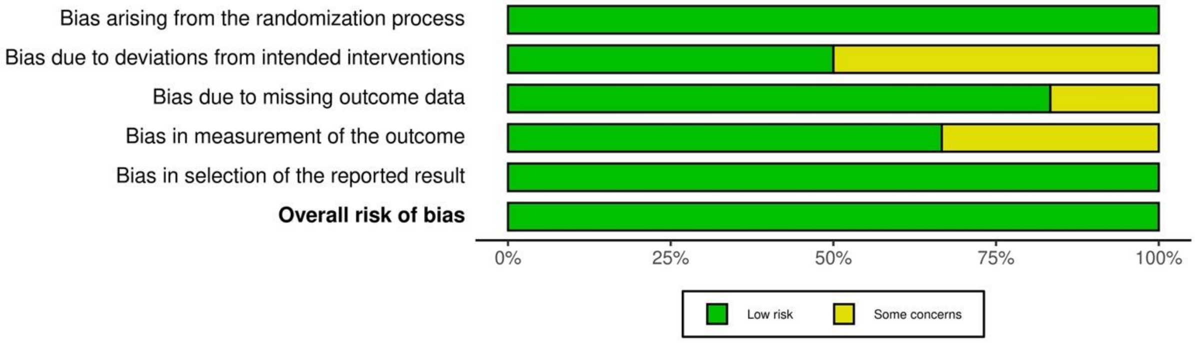

Figure S2. A plot of the distribution of review authors' judgements across randomized studies for each risk of bias item.

Risk of bias domains

Study	D1	D2	D3	D4	D5	D6	D7	Overall
Aselmann et al. 2018	+	-	-	+	+	+	+	+
Asklid et al. 2018	-	-	+	X	+	-	+	-
Baek et al. 2010	-	-	-	-	-	+	-	-
Baek et al. 2012	-	+	+	-	X	-	+	-
Baek et al. 2013	+	-	+	+	-	+	+	+
Barnajian et al. 2014	+	X	-	+	+	+	-	-
Bedirli et al. 2015	-	+	-	+	+	-	+	+
Bedrikovetski et al. 2020	-	-	+	+	-	-	+	-
Bianchi et al. 2010	-	-	-	+	+	+	+	+
Bilgin et al. 2020	-	-	-	-	+	+	+	-
Chen et al. 2017	-	X	+	-	-	+	-	-
Cho et al. 2015	-	X	-	-	+	-	+	-
Crolla et al. 2018	+	-	+	+	+	+	-	+
D'Annibale et al. 2013	+	+	+	+	-	+	-	+
De Jesus et al. 2016	-	+	-	+	-	+	+	+
De'Angelis et al. 2020	-	+	+	-	+	+	+	+
Esen et al. 2018	-	+	-	+	-	+	+	+
Feroci et al. 2016	+	+	+	-	-	+	-	+
Garfinkle et al. 2019	+	+	+	+	-	-	+	+
Ishihara et al. 2018	-	+	+	+	-	+	+	+
Kang et al. 2013	-	+	-	+	-	+	+	+
Kethman et al. 2020	+	-	-	+	-	+	+	+
Kim et al. 2012	-	-	+	+	+	+	+	+
Kim et al. 2016	-	-	+	+	+	+	+	+
Law et al. 2016	-	+	+	+	-	+	+	+
Lim et al. 2016	-	-	-	+	+	+	-	-
Liu et al. 2019	+	-	+	+	+	-	-	+
Park et al. 2011	-	+	+	+	+	-	+	+
Ramji et al. 2016	+	+	-	+	+	-	+	+
Rouanet et al. 2018	+	+	+	-	+	-	+	+
Shiomi et al. 2016	-	+	-	+	-	-	-	-
Silva-Velazco et al. 2017	-	+	-	-	+	+	+	+
Sugoor et al. 2018	+	+	+	+	-	+	+	+
Valverde et al. 2017	-	+	-	+	-	+	+	+
Yamaguchi et al. 2016	+	+	+	+	-	-	+	+

Domains:
D1: Bias due to confounding.
D2: Bias due to selection of participants.
D3: Bias in classification of interventions.
D4: Bias due to deviations from intended interventions.
D5: Bias due to missing data.
D6: Bias in measurement of outcomes.
D7: Bias in selection of the reported result.

Judgement
X Serious
- Moderate
+ Low

Figure S3. A summary table of review authors' judgements for each risk of bias item for each non-randomized study.

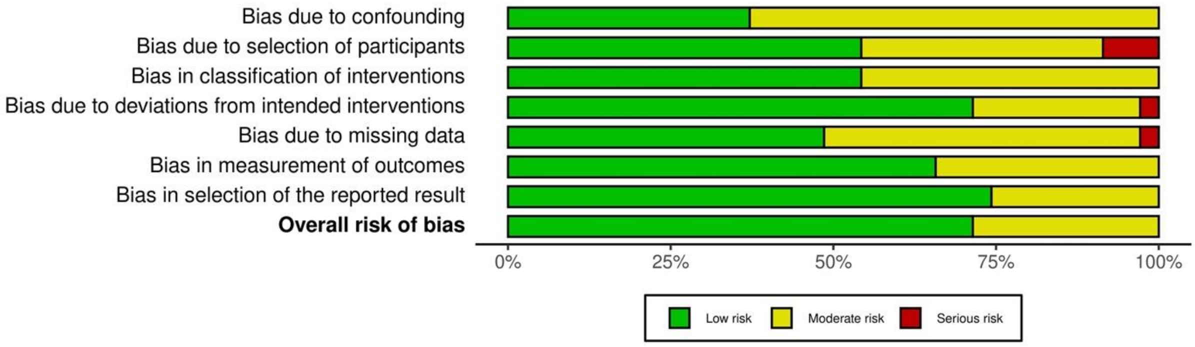

Figure S4. A plot of the distribution of review authors' judgements across non-randomized studies for each risk of bias item.

	Robotic-Assisted			Laparoscopic				Mean Difference
Study or Subgroup	Mean	SD	Total	Mean	SD	Total	Weight	IV, Random, 95% CI
Ahmed 2017	243.8	14.2	99	272.5	18.3	85	3.2%	-28.70 [-33.49, -23.91]
Aselmann 2018	280	76	44	199	81	41	2.9%	81.00 [47.55, 114.45]
Baek 2010	315.5	106.8	41	347	118.4	41	2.6%	-31.50 [-80.31, 17.31]
Baek 2012	285.2	69.1	154	219.7	71.2	150	3.1%	65.50 [49.72, 81.28]
Baek 2013	352.7	130.3	47	360.7	88.2	37	2.6%	-8.00 [-54.85, 38.85]
Baik 2008	217.1	51.6	18	204.3	51.9	16	2.8%	12.80 [-22.06, 47.66]
Barnajian 2014	292.5	112.6	20	245	98.2	20	2.2%	47.50 [-17.98, 112.98]
Bedirli 2015	252	62	35	208	49	28	2.9%	44.00 [16.59, 71.41]
Bianchi 2010	267.5	72.2	25	297.3	108.3	25	2.5%	-29.80 [-80.82, 21.22]
Bilgin 2020	341.9	111.7	72	262.7	97.5	44	2.8%	79.20 [40.53, 117.87]
Cho 2015	361.6	91.9	278	272.4	83.8	278	3.1%	89.20 [74.58, 103.82]
Crolla 2018	219	47.4	168	172	48.4	184	3.1%	47.00 [36.99, 57.01]
de'Angelis 2020	245	46.2	44	210	46.2	44	3.1%	35.00 [15.69, 54.31]
D'Annibale 2013	273.8	21.7	50	287.5	31.8	50	3.1%	-13.70 [-24.37, -3.03]
Feroci 2016	367.3	82.9	53	202.3	70.7	53	2.9%	165.00 [135.67, 194.33]
Garfinkle 2019	295.6	115.4	154	296.3	117.5	213	3.0%	-0.70 [-24.81, 23.41]
Ishihara 2018	397.5	37.7	130	284.8	24.2	318	3.1%	112.70 [105.69, 119.71]
Jayne 2017	298.5	88.71	236	261	83.24	230	3.1%	37.50 [21.89, 53.11]
Kang 2013	309.7	115.2	165	277.8	81.9	165	3.0%	31.90 [10.33, 53.47]
Kethman 2020	299.16	119.1	192	304	123.7	206	3.0%	-4.84 [-28.70, 19.02]
Kim 2016	441	90.2	33	277	83.2	66	2.8%	164.00 [127.26, 200.74]
Kim 2018	339.2	80.1	66	227.8	65.6	73	3.0%	111.40 [86.91, 135.89]
Law 2016	332	89	220	267.8	69.8	171	3.1%	64.20 [48.46, 79.94]
Lim 2016	365.2	108.4	74	311.6	79.8	64	2.9%	53.60 [22.10, 85.10]
Liu 2019	206.5	59.08	80	201.68	50.49	116	3.1%	4.82 [-11.06, 20.70]
Park 2011	232.6	52.4	52	158.1	49.2	123	3.1%	74.50 [57.81, 91.19]
Patriti 2009	202	12	29	208	7	37	3.2%	-6.00 [-10.92, -1.08]
Ramji 2016	407	97	26	240	89	27	2.5%	167.00 [116.83, 217.17]
Rouanet 2018	248.3	61.2	200	249.8	54.8	200	3.1%	-1.50 [-12.89, 9.89]
Shiomi 2016	269.8	60.2	127	255	49.3	109	3.1%	14.80 [0.83, 28.77]
Silva-Velazco 2017	315.3	116.3	66	269.8	68.2	118	2.9%	45.50 [14.86, 76.14]
Sugoor 2018	372.4	102.8	84	301	53.6	84	3.0%	71.40 [46.61, 96.19]
Valverde 2017	215	53	65	226	66	65	3.0%	-11.00 [-31.58, 9.58]
Yamaguchi 2016	232.9	72	203	227.6	62.6	239	3.1%	5.30 [-7.39, 17.99]
**Total (95% CI)**			**3350**			**3720**	**100.0%**	**43.39 [25.26, 61.51]**

Heterogeneity: $Tau^2 = 2707.12$; $Chi^2 = 1776.95$, df = 33 ($P < 0.00001$); $I^2 = 98\%$
Test for overall effect: Z = 4.69 ($P < 0.00001$)

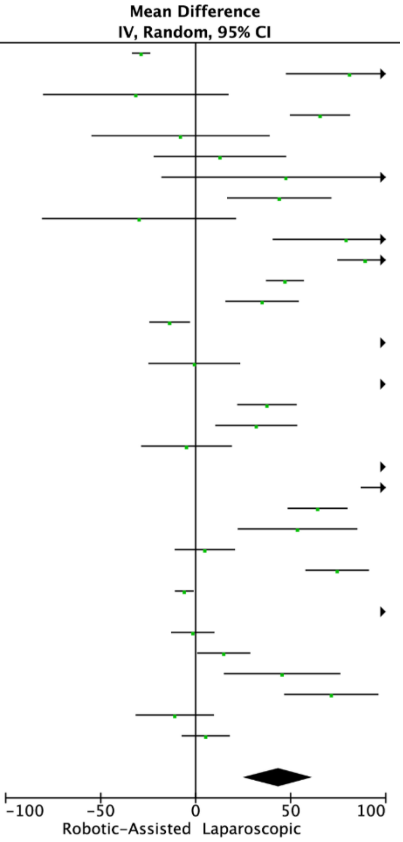

Figure S5. Forest plot of operative time in the robotic-assisted and laparoscopic groups. The center of each square represents the weighted mean difference for individual trials, and the corresponding horizontal line stands for a 95% confidence interval. The diamonds represent pooled results. Legend: CI = confidence interval.

Study or Subgroup	Robotic-Assisted Events	Robotic-Assisted Total	Laparoscopic Events	Laparoscopic Total	Weight	Odds Ratio M-H, Random, 95% CI
Ahmed 2017	0	99	4	85	0.9%	0.09 [0.00, 1.72]
Aselmann 2018	3	44	7	41	3.8%	0.36 [0.09, 1.48]
Asklid 2018	8	72	16	47	8.5%	0.24 [0.09, 0.63]
Baek 2010	3	41	9	41	4.0%	0.28 [0.07, 1.13]
Baek 2013	1	47	6	37	1.6%	0.11 [0.01, 0.98]
Baik 2008	0	18	2	16	0.8%	0.16 [0.01, 3.53]
Barnajian 2014	0	20	2	20	0.8%	0.18 [0.01, 4.01]
Bianchi 2010	0	25	1	25	0.7%	0.32 [0.01, 8.25]
Bilgin 2020	1	72	0	44	0.7%	1.87 [0.07, 46.84]
Cho 2015	1	278	2	278	1.3%	0.50 [0.04, 5.53]
Corrigan 2018	19	236	28	230	20.4%	0.63 [0.34, 1.17]
Crolla 2018	3	168	23	184	5.1%	0.13 [0.04, 0.43]
D'Annibale 2013	0	50	6	50	0.9%	0.07 [0.00, 1.24]
Esen 2018	2	100	1	78	1.3%	1.57 [0.14, 17.65]
Garfinkle 2019	10	154	32	213	13.9%	0.39 [0.19, 0.83]
Ishihara 2018	0	130	6	318	0.9%	0.18 [0.01, 3.29]
Kang 2013	1	165	3	165	1.5%	0.33 [0.03, 3.20]
Kim 2016	2	33	0	66	0.8%	10.56 [0.49, 226.44]
Kim 2018	1	66	0	73	0.7%	3.37 [0.13, 84.08]
Law 2016	2	220	6	171	2.9%	0.25 [0.05, 1.27]
Lim 2016	1	74	4	64	1.6%	0.21 [0.02, 1.89]
Liu 2019	1	80	4	116	1.6%	0.35 [0.04, 3.23]
Park 2011	0	52	0	123		Not estimable
Patriti 2009	0	29	7	37	0.9%	0.07 [0.00, 1.26]
Ramji 2016	3	26	10	27	3.7%	0.22 [0.05, 0.93]
Rouanet 2018	4	200	19	200	6.4%	0.19 [0.06, 0.58]
Silva-Velazco 2017	6	66	18	118	8.0%	0.56 [0.21, 1.48]
Sugoor 2018	1	84	1	84	1.0%	1.00 [0.06, 16.26]
Valverde 2017	3	65	11	65	4.3%	0.24 [0.06, 0.90]
Yamaguchi 2016	0	203	8	239	0.9%	0.07 [0.00, 1.17]
**Total (95% CI)**		**2917**		**3255**	**100.0%**	**0.35 [0.26, 0.46]**
Total events	76		236			

Heterogeneity: $Tau^2 = 0.00$; $Chi^2 = 26.20$, df = 28 (P = 0.56); $I^2 = 0\%$
Test for overall effect: Z = 7.51 (P < 0.00001)

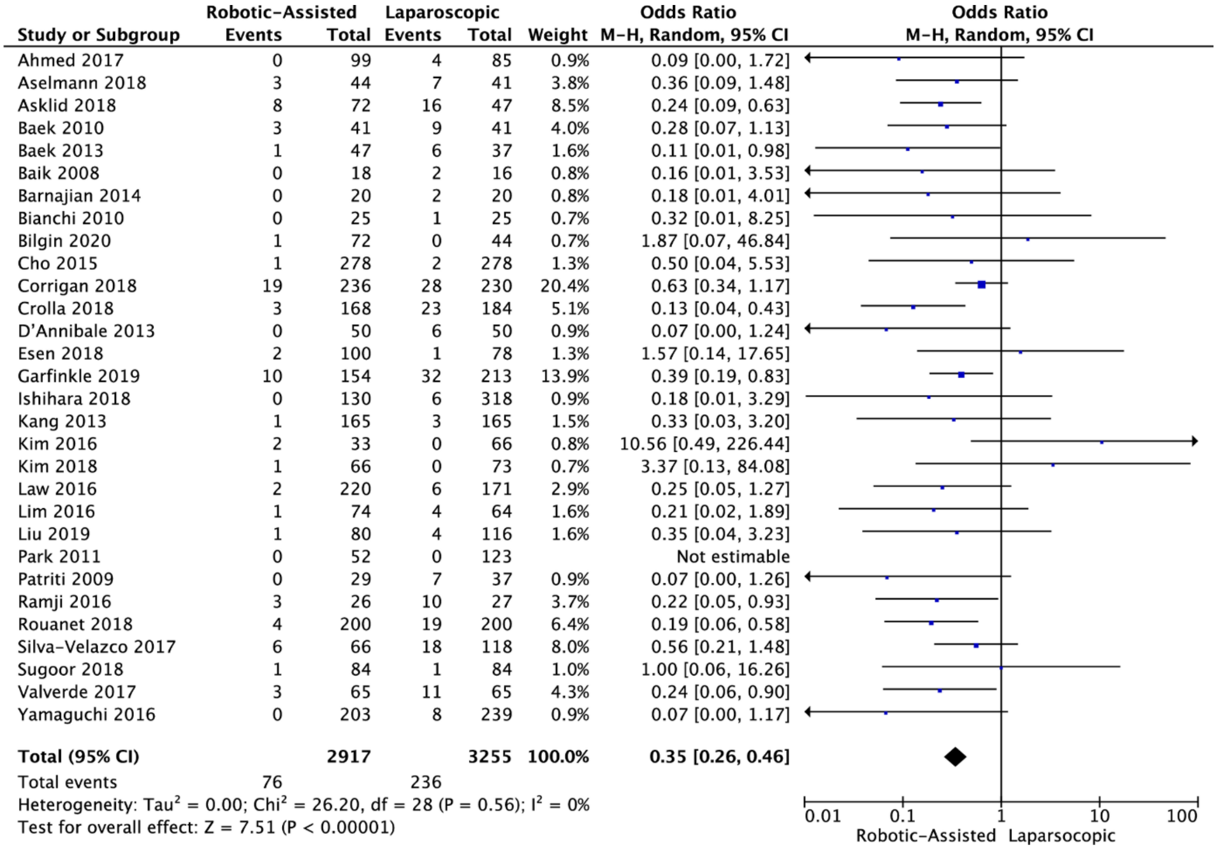

Figure S6. Forest plot of conversion to open surgery rate in the robotic-assisted and laparoscopic groups. The center of each square represents the weighted odds ratio for individual trials, and the corresponding horizontal line stands for a 95% confidence interval. The diamonds represent pooled results. Legend: CI = confidence interval.

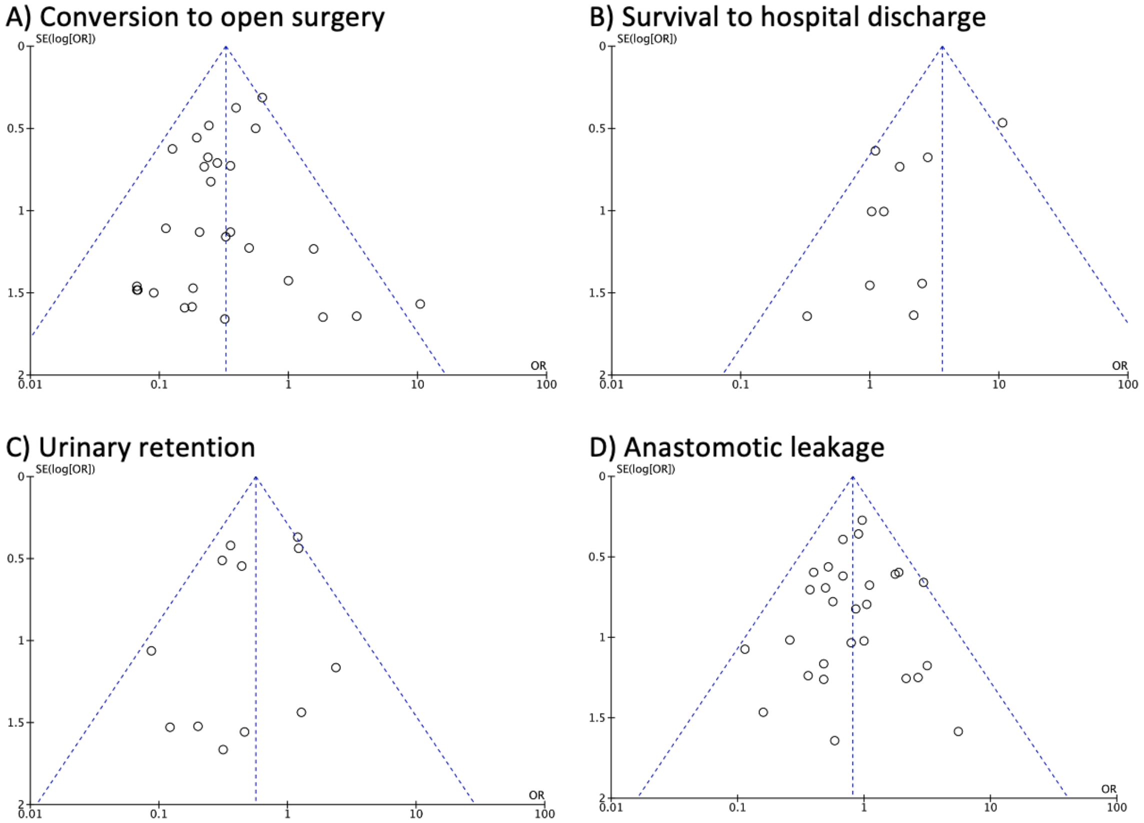

Figure S7. Funnel plot with 95% confidence limits for publication bias in the studies investigating: (A) conversion to open surgery, (B) survival to hospital discharge, (C) urinary retention occurrence, (D) anastomotic leakage occurrence.

	Robotic-Assisted			Laparoscopic				Mean Difference
Study or Subgroup	Mean	SD	Total	Mean	SD	Total	Weight	IV, Random, 95% CI
Ahmed 2017	8.1	2.1	99	105.6	33.8	85	5.3%	-97.50 [-104.70, -90.30]
Baek 2010	605	571.6	41	404.3	283.8	41	1.6%	200.70 [5.36, 396.04]
Baek 2012	167.8	26.1	154	126	267.7	150	4.8%	41.80 [-1.24, 84.84]
Baek 2013	190.9	284.7	47	302.7	305.3	37	2.6%	-111.80 [-239.48, 15.88]
Barnajian 2014	237.5	173.2	20	325	245.4	20	2.6%	-87.50 [-219.14, 44.14]
Bedirli 2015	120	15	35	165	40	28	5.3%	-45.00 [-60.63, -29.37]
Cho 2015	179	236.5	278	147	295.3	278	4.8%	32.00 [-12.47, 76.47]
de'Angelis 2020	130	57.7	44	163.8	73.6	44	5.1%	-33.80 [-61.43, -6.17]
Esen 2018	134	62	100	83	46	78	5.3%	51.00 [35.13, 66.87]
Feroci 2016	130.4	115.5	53	123.7	115.5	58	4.8%	6.70 [-36.32, 49.72]
Ishihara 2018	56	23	130	36.3	15.7	318	5.3%	19.70 [15.39, 24.01]
Kang 2013	133	192.3	165	140.1	216.4	165	4.8%	-7.10 [-51.27, 37.07]
Kim 2016	232	180	33	205	163.8	66	4.0%	27.00 [-46.03, 100.03]
Kim 2018	300	288.7	66	100	86.6	73	4.0%	200.00 [127.57, 272.43]
Law 2016	677.5	415	220	552.5	331.7	171	4.0%	125.00 [50.98, 199.02]
Liu 2019	175.06	110.77	80	123.91	99.61	116	5.1%	51.15 [20.86, 81.44]
Patriti 2009	137.4	156	29	127	169	37	3.9%	10.40 [-68.27, 89.07]
Ramji 2016	296	155	26	524	501	27	1.5%	-228.00 [-426.14, -29.86]
Rouanet 2018	300	133.3	200	450	266.7	200	4.8%	-150.00 [-191.32, -108.68]
Shiomi 2016	39.5	23	127	129.8	81.5	109	5.3%	-90.30 [-106.11, -74.49]
Silva-Velazco 2017	876.3	856	66	853.8	497.5	118	1.3%	22.50 [-202.68, 247.68]
Sugoor 2018	213.5	251.1	84	208.5	215.5	84	4.1%	5.00 [-65.76, 75.76]
Valverde 2017	143	180	65	92	112	65	4.6%	51.00 [-0.54, 102.54]
Yamaguchi 2016	15.4	26.4	203	39.1	85.1	239	5.3%	-23.70 [-35.08, -12.32]
**Total (95% CI)**			**2365**			**2607**	**100.0%**	**-0.94 [-30.11, 28.22]**

Heterogeneity: $Tau^2 = 4138.44$; $Chi^2 = 1063.61$, df = 23 (P < 0.00001); $I^2 = 98\%$
Test for overall effect: Z = 0.06 (P = 0.95)

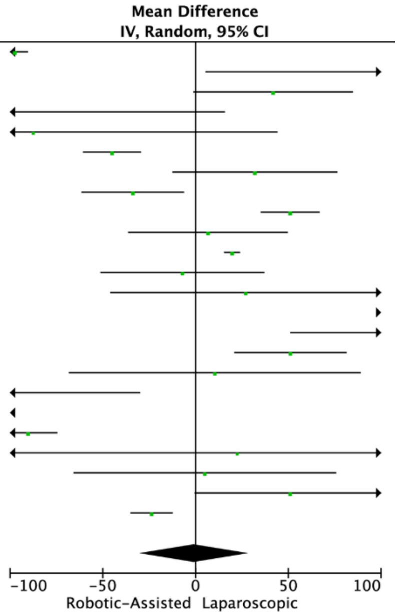

Figure S8. Forest plot of intraoperative blood loss in the robotic-assisted and laparoscopic groups. The center of each square represents the weighted mean difference for individual trials, and the corresponding horizontal line stands for a 95% confidence interval. The diamonds represent pooled results. Legend: CI = confidence interval.

Study or Subgroup	Robotic-Assisted Mean	SD	Total	Laparoscopic Mean	SD	Total	Weight	Mean Difference IV, Random, 95% CI
Ahmed 2017	17	1.7	99	18.3	1.8	85	4.1%	−1.30 [−1.81, −0.79]
Aselmann 2018	21.2	9.5	44	20	7	41	2.7%	1.20 [−2.33, 4.73]
Asklid 2018	19.9	9.3	72	16.7	8.4	47	2.9%	3.20 [−0.02, 6.42]
Baek 2010	15.6	8.7	41	19.1	9.8	41	2.5%	−3.50 [−7.51, 0.51]
Baek 2013	10.6	6.3	47	14.1	10.4	37	2.6%	−3.50 [−7.30, 0.30]
Baik 2008	20	9.1	18	17.4	10.6	16	1.5%	2.60 [−4.08, 9.28]
Barnajian 2014	13.3	5.5	20	11	4	20	3.0%	2.30 [−0.68, 5.28]
Bedirli 2015	27	11	35	23	8	28	2.2%	4.00 [−0.70, 8.70]
Bedrikovetski 2020	20	7.5	117	28.3	13.8	1269	3.7%	−8.30 [−9.86, −6.74]
Bianchi 2010	19.3	7.8	25	19.8	8.4	25	2.3%	−0.50 [−4.99, 3.99]
Bilgin 2020	28.3	15.9	72	23.5	8.7	44	2.3%	4.80 [0.32, 9.28]
Cho 2015	15	8.1	278	16.2	8.1	278	3.8%	−1.20 [−2.55, 0.15]
Crolla 2018	18.5	7	168	14.5	7.3	184	3.8%	4.00 [2.51, 5.49]
de Jesus 2016	16.3	13	59	20.8	15.9	41	1.7%	−4.50 [−10.39, 1.39]
de'Angelis 2020	15.9	5.39	44	16.93	6.58	44	3.3%	−1.03 [−3.54, 1.48]
D'Annibale 2013	16.5	7.1	50	13.8	6.7	50	3.2%	2.70 [−0.01, 5.41]
Esen 2018	25	12	100	27	11	78	2.8%	−2.00 [−5.39, 1.39]
Feroci 2016	22.3	13	53	13	6.8	58	2.5%	9.30 [5.39, 13.21]
Garfinkle 2019	15.6	7.6	154	16.6	7.4	213	3.7%	−1.00 [−2.56, 0.56]
Jayne 2017	23.2	11.97	235	24.1	12.9	221	3.4%	−0.90 [−3.19, 1.39]
Kang 2013	15	9.4	165	15.6	9.1	165	3.5%	−0.60 [−2.60, 1.40]
Kim 2016	22.3	11.7	33	21.6	11	66	2.1%	0.70 [−4.09, 5.49]
Kim 2018	25	15	66	18.5	10.4	73	2.3%	6.50 [2.17, 10.83]
Lim 2016	11.6	6.9	74	14.7	6.5	64	3.4%	−3.10 [−5.34, −0.86]
Liu 2019	11.71	5.19	80	12.26	4.85	116	3.8%	−0.55 [−1.99, 0.89]
Park 2011	19.4	10.2	52	15.9	10.1	123	2.9%	3.50 [0.20, 6.80]
Patriti 2009	10.3	4	29	11.2	5	37	3.5%	−0.90 [−3.07, 1.27]
Ramji 2016	16.7	6.8	26	16.8	7.7	27	2.5%	−0.10 [−4.01, 3.81]
Rouanet 2018	26.8	12.5	200	29.8	12.8	200	3.3%	−3.00 [−5.48, −0.52]
Shiomi 2016	30.8	8.2	127	30.5	0.3	109	3.8%	0.30 [−1.13, 1.73]
Silva-Velazco 2017	39.3	28.6	66	45	21	118	1.2%	−5.70 [−13.57, 2.17]
Sugoor 2018	14.1	9.5	84	14.6	9.2	84	3.1%	−0.50 [−3.33, 2.33]
Valverde 2017	17	9	65	19	10	65	2.9%	−2.00 [−5.27, 1.27]
Yamaguchi 2016	30	10.3	203	29.3	11.8	239	3.5%	0.70 [−1.36, 2.76]
**Total (95% CI)**			**3001**			**4306**	**100.0%**	**−0.05 [−1.06, 0.96]**

Heterogeneity: $Tau^2 = 6.47$; $Chi^2 = 224.50$, df = 33 ($P < 0.00001$); $I^2 = 85\%$
Test for overall effect: Z = 0.10 (P = 0.92)

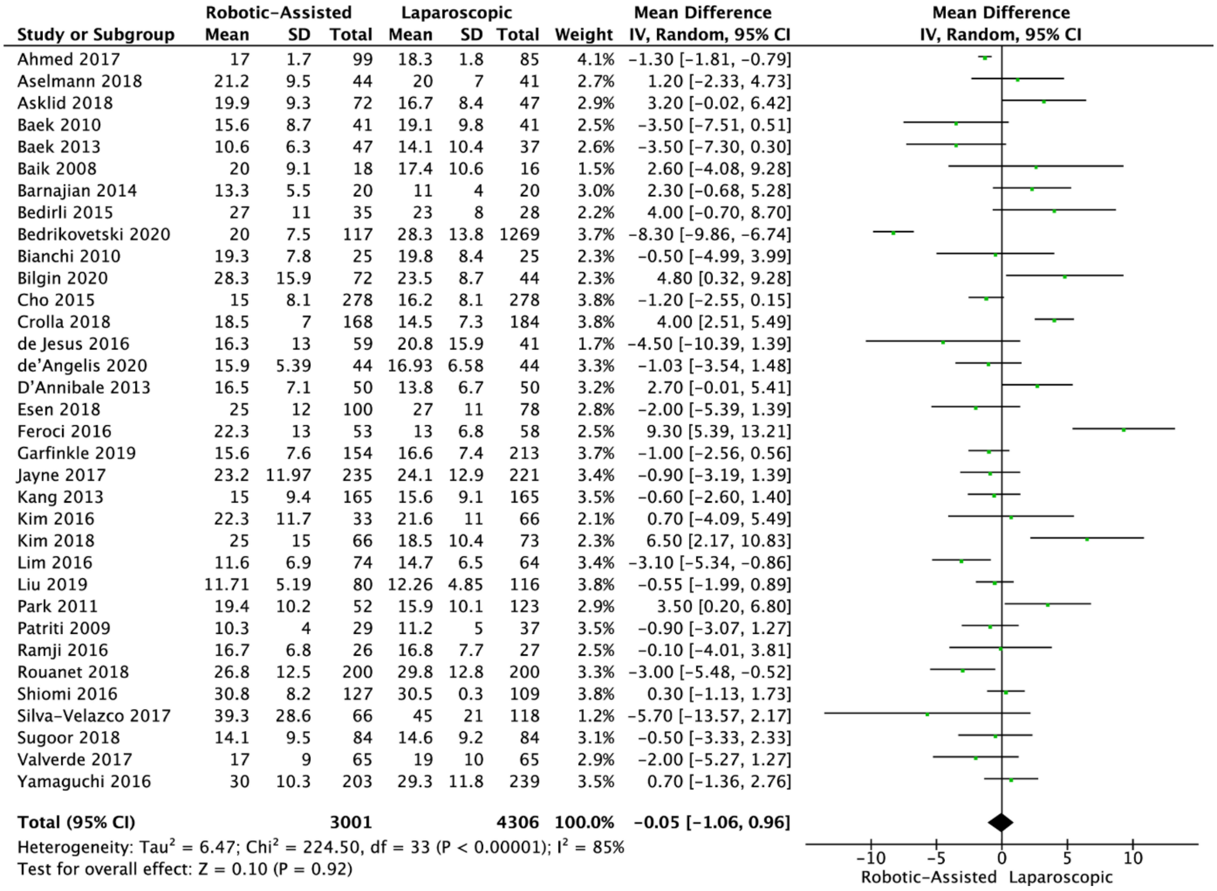

Figure S9. Forest plot of lymph nodes harvested in the robotic-assisted and laparoscopic groups. The center of each square represents the weighted mean difference for individual trials, and the corresponding horizontal line stands for a 95% confidence interval. The diamonds represent pooled results. Legend: CI = confidence interval.

Study or Subgroup	Robotic-Assisted Events	Total	Laparoscopic Events	Total	Weight	Odds Ratio M-H, Random, 95% CI
Ahmed 2017	99	99	85	85		Not estimable
Aselmann 2018	44	44	41	41		Not estimable
Asklid 2018	72	72	47	47		Not estimable
Baek 2010	41	41	41	41		Not estimable
Barnajian 2014	19	20	19	20	5.4%	1.00 [0.06, 17.18]
Bedrikovetski 2020	117	117	1256	1269	5.5%	2.52 [0.15, 42.74]
Bilgin 2020	68	72	40	44	13.3%	1.70 [0.40, 7.17]
Chen 2017	4739	4744	5516	5578	18.9%	10.65 [4.28, 26.52]
Crolla 2018	165	168	175	184	14.4%	2.83 [0.75, 10.63]
de'Angelis 2020	44	44	44	44		Not estimable
Feroci 2016	48	53	52	58	15.1%	1.11 [0.32, 3.87]
Garfinkle 2019	154	154	212	213	4.5%	2.18 [0.09, 53.90]
Jayne 2017	234	236	228	230	9.3%	1.03 [0.14, 7.35]
Kang 2013	165	165	165	165		Not estimable
Law 2016	218	220	169	171	9.3%	1.29 [0.18, 9.25]
Patriti 2009	29	29	37	37		Not estimable
Ramji 2016	26	26	27	27		Not estimable
Valverde 2017	64	65	65	65	4.5%	0.33 [0.01, 8.21]
**Total (95% CI)**		**6369**		**8319**	**100.0%**	**2.10 [1.00, 4.43]**
Total events	6346		8219			

Heterogeneity: $Tau^2 = 0.55$; $Chi^2 = 15.72$, df = 9 ($P = 0.07$); $I^2 = 43\%$
Test for overall effect: Z = 1.96 (P = 0.05)

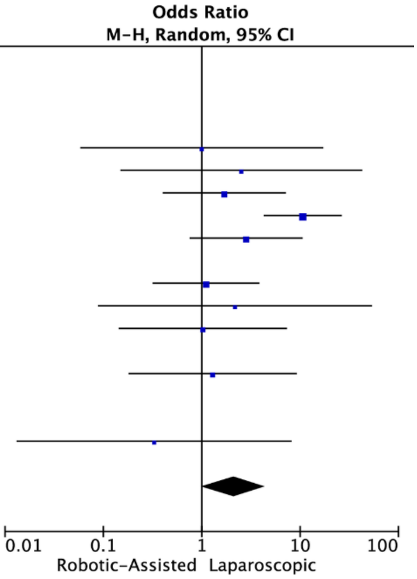

Figure S10. Forest plot of survival to hospital discharge in the robotic-assisted and laparoscopic groups. The center of each square represents the weighted odds ratio for individual trials, and the corresponding horizontal line stands for a 95% confidence interval. The diamonds represent pooled results. Legend: CI = confidence interval.

Study or Subgroup	Robotic-Assisted Mean	Robotic-Assisted SD	Robotic-Assisted Total	Laparoscopic Mean	Laparoscopic SD	Laparoscopic Total	Weight	Mean Difference IV, Random, 95% CI
Ahmed 2017	8	1.3	99	10	1.3	85	3.4%	−2.00 [−2.38, −1.62]
Baek 2010	12	9	41	9.1	4.9	41	2.4%	2.90 [−0.24, 6.04]
Baek 2012	11.1	7	154	10.8	8.6	150	3.0%	0.30 [−1.47, 2.07]
Baek 2013	9.8	2	47	12.4	2.8	37	3.3%	−2.60 [−3.67, −1.53]
Baik 2008	6.9	1.3	18	8.7	1.3	16	3.3%	−1.80 [−2.68, −0.92]
Barnajian 2014	11.8	7.8	20	13.8	9	20	1.6%	−2.00 [−7.22, 3.22]
Bedirli 2015	5.1	3.7	35	4.6	2.8	28	3.1%	0.50 [−1.11, 2.11]
Bedrikovetski 2020	12.8	4.8	117	29.5	16.3	1269	3.2%	−16.70 [−17.95, −15.45]
Bianchi 2010	8	3.2	25	9	4.7	25	2.8%	−1.00 [−3.23, 1.23]
Bilgin 2020	5	2.4	72	9.5	5.2	44	3.1%	−4.50 [−6.13, −2.87]
Chen 2017	6.4	0.8	4744	4.8	0.6	5578	3.4%	1.60 [1.57, 1.63]
Cho 2015	10.4	5.6	278	10.7	6.6	278	3.3%	−0.30 [−1.32, 0.72]
Crolla 2018	20.3	10.8	168	30.3	16.8	184	2.5%	−10.00 [−12.93, −7.07]
de'Angelis 2020	7.25	3.37	44	7.64	3.45	44	3.2%	−0.39 [−1.82, 1.04]
D'Annibale 2013	8.5	1.2	50	10.5	1.7	50	3.4%	−2.00 [−2.58, −1.42]
Esen 2018	7	4	100	9	4	78	3.2%	−2.00 [−3.18, −0.82]
Feroci 2016	8	4.1	53	18.5	13.9	58	2.2%	−10.50 [−14.24, −6.76]
Garfinkle 2019	4	0.6	154	5.3	0.9	213	3.4%	−1.30 [−1.45, −1.15]
Jayne 2017	8	5.85	223	8.2	6.03	221	3.3%	−0.20 [−1.31, 0.91]
Kang 2013	10.8	5.5	165	13.5	9.2	165	3.1%	−2.70 [−4.34, −1.06]
Kethman 2020	5.9	6	192	6.8	7.7	206	3.2%	−0.90 [−2.25, 0.45]
Kim 2016	10.9	6.2	33	13.1	12.8	66	2.2%	−2.20 [−5.94, 1.54]
Kim 2018	10.3	3.4	66	10.8	7.4	73	3.0%	−0.50 [−2.39, 1.39]
Lim 2016	8	6	872	8	6	4737	3.4%	0.00 [−0.43, 0.43]
Liu 2019	11.2	5.8	80	14.72	6.9	116	3.0%	−3.52 [−5.31, −1.73]
Park 2011	10.4	4.7	52	9.8	3.8	123	3.2%	0.60 [−0.84, 2.04]
Patriti 2009	11.9	7.5	29	9.6	6.9	37	2.2%	2.30 [−1.22, 5.82]
Ramji 2016	7	3.4	26	11.3	13.7	27	1.6%	−4.30 [−9.63, 1.03]
Rouanet 2018	23.3	10.8	200	21	9.7	200	2.9%	2.30 [0.29, 4.31]
Shiomi 2016	12.3	3.8	125	16.5	6.3	109	3.2%	−4.20 [−5.56, −2.84]
Silva−Velazco 2017	10	7.5	66	12	5	118	2.9%	−2.00 [−4.02, 0.02]
Sugoor 2018	7.2	3.4	84	8.1	4.7	84	3.2%	−0.90 [−2.14, 0.34]
Valverde 2017	11	8	65	12	10	65	2.4%	−1.00 [−4.11, 2.11]
Yamaguchi 2016	7.3	2.3	203	9.3	6.7	239	3.3%	−2.00 [−2.91, −1.09]
**Total (95% CI)**			**8700**			**14784**	**100.0%**	**−2.01 [−2.90, −1.11]**

Heterogeneity: $Tau^2 = 6.10$; $Chi^2 = 3213.73$, df = 33 ($P < 0.00001$); $I^2 = 99\%$
Test for overall effect: Z = 4.38 ($P < 0.0001$)

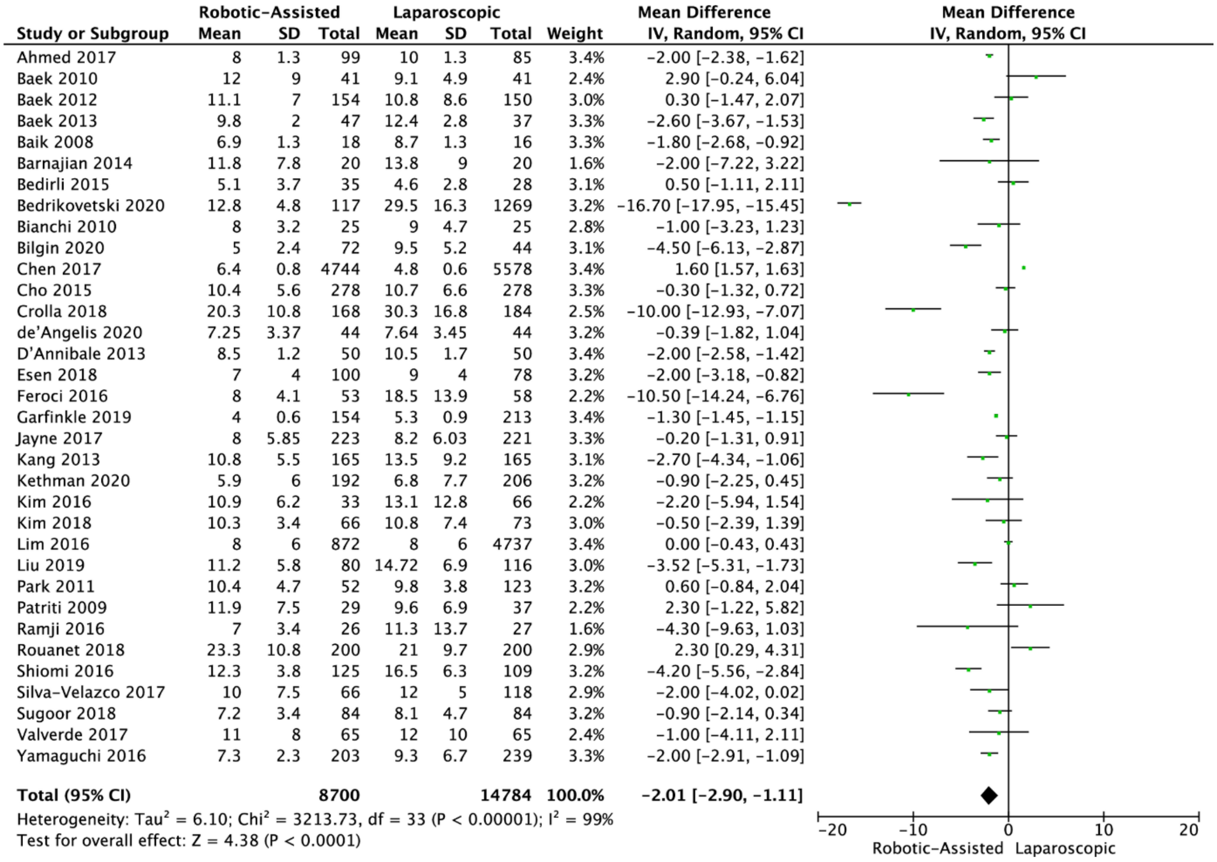

Figure S11. Forest plot of hospital length of stay in the robotic-assisted and laparoscopic groups. The center of each square represents the weighted mean difference for individual trials, and the corresponding horizontal line stands for a 95% confidence interval. The diamonds represent pooled results. Legend: CI = confidence interval.

Study or Subgroup	Robotic-Assisted Events	Robotic-Assisted Total	Laparoscopic Events	Laparoscopic Total	Weight	Odds Ratio M−H, Random, 95% CI
Aselmann 2018	0	44	3	41	2.6%	0.12 [0.01, 2.47]
Barnajian 2014	0	20	1	20	2.2%	0.32 [0.01, 8.26]
Cho 2015	5	278	11	278	12.9%	0.44 [0.15, 1.30]
Esen 2018	3	100	1	78	4.2%	2.38 [0.24, 23.35]
Jayne 2017	17	236	14	230	18.5%	1.20 [0.58, 2.49]
Law 2016	9	220	18	171	16.7%	0.36 [0.16, 0.83]
Liu 2019	0	80	3	116	2.6%	0.20 [0.01, 3.95]
Park 2011	0	52	2	123	2.5%	0.46 [0.02, 9.81]
Patriti 2009	1	29	1	37	2.9%	1.29 [0.08, 21.47]
Shiomi 2016	1	127	9	109	4.9%	0.09 [0.01, 0.71]
Silva−Velazco 2017	10	66	15	118	16.1%	1.23 [0.52, 2.91]
Yamaguchi 2016	5	203	18	239	13.7%	0.31 [0.11, 0.85]
**Total (95% CI)**		**1455**		**1560**	**100.0%**	**0.56 [0.34, 0.92]**
Total events	51		96			

Heterogeneity: $Tau^2 = 0.22$; $Chi^2 = 16.30$, df = 11 ($P = 0.13$); $I^2 = 33\%$
Test for overall effect: Z = 2.27 ($P = 0.02$)

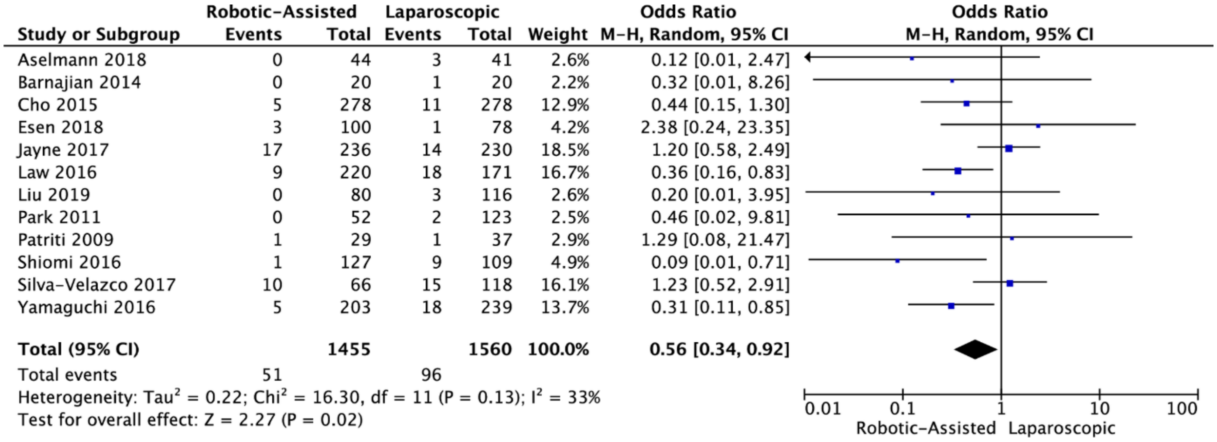

Figure S12. Forest plot of urinary retention rate in the robotic-assisted and laparoscopic groups. The center of each square represents the weighted odds ratio for individual trials, and the corresponding horizontal line stands for a 95% confidence interval. The diamonds represent pooled results. Legend: CI = confidence interval.

Study or Subgroup	Robotic-Assisted Events	Robotic-Assisted Total	Laparoscopic Events	Laparoscopic Total	Weight	Odds Ratio M-H, Random, 95% CI
Aselmann 2018	2	44	2	41	7.0%	0.93 [0.12, 6.92]
Bedrikovetski 2020	7	117	31	1269	39.5%	2.54 [1.09, 5.90]
Cho 2015	16	278	9	278	40.3%	1.83 [0.79, 4.20]
de'Angelis 2020	1	44	0	44	2.7%	3.07 [0.12, 77.41]
Silva-Velazco 2017	2	66	6	118	10.6%	0.58 [0.11, 2.98]
**Total (95% CI)**		**549**		**1750**	**100.0%**	**1.78 [1.05, 3.03]**
Total events	28		48			

Heterogeneity: $Tau^2 = 0.00$; $Chi^2 = 3.05$, df = 4 (P = 0.55); $I^2 = 0\%$
Test for overall effect: Z = 2.14 (P = 0.03)

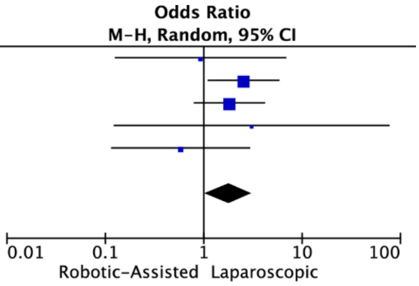

Figure S13. Forest plot of bowel obstruction rate in the robotic-assisted and laparoscopic groups. The center of each square represents the weighted odds ratio for individual trials, and the corresponding horizontal line stands for a 95% confidence interval. The diamonds represent pooled results. Legend: CI = confidence interval.

Study or Subgroup	Robotic-Assisted Events	Robotic-Assisted Total	Laparoscopic Events	Laparoscopic Total	Weight	Odds Ratio M-H, Random, 95% CI
Ahmed 2017	1	85	2	63	1.0%	0.36 [0.03, 4.10]
Aselmann 2018	5	44	10	41	4.4%	0.40 [0.12, 1.28]
Baek 2010	3	41	1	41	1.1%	3.16 [0.31, 31.70]
Baek 2012	17	154	18	150	12.2%	0.91 [0.45, 1.84]
Baek 2013	4	47	3	37	2.5%	1.05 [0.22, 5.03]
Barnajian 2014	2	20	0	20	0.6%	5.54 [0.25, 123.08]
Bedirli 2015	2	35	2	28	1.5%	0.79 [0.10, 5.98]
Bedrikovetski 2020	1	117	41	1269	1.5%	0.26 [0.04, 1.89]
Bianchi 2010	1	25	2	25	1.0%	0.48 [0.04, 5.65]
Cho 2015	29	278	30	278	20.7%	0.96 [0.56, 1.65]
Crolla 2018	5	112	10	122	4.9%	0.52 [0.17, 1.58]
de'Angelis 2020	2	44	2	44	1.5%	1.00 [0.13, 7.43]
D'Annibale 2013	5	50	7	50	4.1%	0.68 [0.20, 2.32]
Esen 2018	3	100	4	78	2.6%	0.57 [0.12, 2.63]
Feroci 2016	3	53	8	58	3.2%	0.38 [0.09, 1.50]
Garfinkle 2019	4	154	5	213	3.4%	1.11 [0.29, 4.20]
Ishihara 2018	0	130	7	318	0.7%	0.16 [0.01, 2.81]
Kang 2013	12	165	17	165	10.1%	0.68 [0.32, 1.48]
Kim 2018	8	66	5	73	4.4%	1.88 [0.58, 6.05]
Law 2016	11	220	3	171	3.6%	2.95 [0.81, 10.74]
Liu 2019	1	80	3	116	1.2%	0.48 [0.05, 4.67]
Park 2011	5	52	7	123	4.2%	1.76 [0.53, 5.83]
Patriti 2009	2	29	1	37	1.0%	2.67 [0.23, 30.96]
Ramji 2016	2	26	1	27	1.0%	2.17 [0.18, 25.46]
Shiomi 2016	3	127	3	109	2.3%	0.85 [0.17, 4.32]
Silva-Velazco 2017	0	66	1	118	0.6%	0.59 [0.02, 14.66]
Sugoor 2018	1	84	8	84	1.4%	0.11 [0.01, 0.94]
Yamaguchi 2016	3	203	7	239	3.2%	0.50 [0.13, 1.95]
**Total (95% CI)**		**2607**		**4097**	**100.0%**	**0.84 [0.65, 1.07]**
Total events	135		208			

Heterogeneity: $Tau^2 = 0.00$; $Chi^2 = 23.47$, df = 27 (P = 0.66); $I^2 = 0\%$
Test for overall effect: Z = 1.41 (P = 0.16)

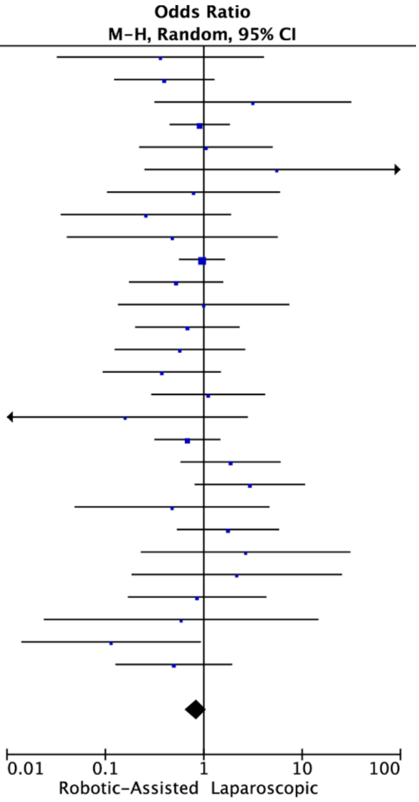

Figure S14. Forest plot of anastomotic leakage rate in the robotic-assisted and laparoscopic groups. The center of each square represents the weighted odds ratio for individual trials, and the corresponding horizontal line stands for a 95% confidence interval. The diamonds represent pooled results. Legend: CI = confidence interval.

Table S1. PRISMA checklist

Section and Topic	Item #	Checklist item	Location where item is reported
**TITLE**			
Title	1	Identify the report as a systematic review.	1
**ABSTRACT**			
Abstract	2	See the PRISMA 2020 for Abstracts checklist.	1
**INTRODUCTION**			
Rationale	3	Describe the rationale for the review in the context of existing knowledge.	2
Objectives	4	Provide an explicit statement of the objective(s) or question(s) the review addresses.	2
**METHODS**			
Eligibility criteria	5	Specify the inclusion and exclusion criteria for the review and how studies were grouped for the syntheses.	3
Information sources	6	Specify all databases, registers, websites, organisations, reference lists and other sources searched or consulted to identify studies. Specify the date when each source was last searched or consulted.	3
Search strategy	7	Present the full search strategies for all databases, registers and websites, including any filters and limits used.	3
Selection process	8	Specify the methods used to decide whether a study met the inclusion criteria of the review, including how many reviewers screened each record and each report retrieved, whether they worked independently, and if applicable, details of automation tools used in the process.	3
Data collection process	9	Specify the methods used to collect data from reports, including how many reviewers collected data from each report, whether they worked independently, any processes for obtaining or confirming data from study investigators, and if applicable, details of automation tools used in the process.	3
Data items	10a	List and define all outcomes for which data were sought. Specify whether all results that were compatible with each outcome domain in each study were sought (e.g. for all measures, time points, analyses), and if not, the methods used to decide which results to collect.	3
	10b	List and define all other variables for which data were sought (e.g. participant and intervention characteristics, funding sources). Describe any assumptions made about any missing or unclear information.	3
Study risk of bias assessment	11	Specify the methods used to assess risk of bias in the included studies, including details of the tool(s) used, how many reviewers assessed each study and whether they worked independently, and if applicable, details of automation tools used in the process.	3
Effect measures	12	Specify for each outcome the effect measure(s) (e.g. risk ratio, mean difference) used in the synthesis or presentation of results.	3
Synthesis methods	13a	Describe the processes used to decide which studies were eligible for each synthesis (e.g. tabulating the study intervention characteristics and comparing against the planned groups for each synthesis (item #5)).	3
	13b	Describe any methods required to prepare the data for presentation or synthesis, such as handling of missing summary statistics, or data conversions.	3
	13c	Describe any methods used to tabulate or visually display results of individual studies and syntheses.	3
	13d	Describe any methods used to synthesize results and provide a rationale for the choice(s). If meta-analysis was performed, describe the	3

Section and Topic	Item #	Checklist item	Location where item is reported
		model(s), method(s) to identify the presence and extent of statistical heterogeneity, and software package(s) used.	
	13e	Describe any methods used to explore possible causes of heterogeneity among study results (e.g. subgroup analysis, meta-regression).	3
	13f	Describe any sensitivity analyses conducted to assess robustness of the synthesized results.	3
Reporting bias assessment	14	Describe any methods used to assess risk of bias due to missing results in a synthesis (arising from reporting biases).	3
Certainty assessment	15	Describe any methods used to assess certainty (or confidence) in the body of evidence for an outcome.	3
**RESULTS**			
Study selection	16a	Describe the results of the search and selection process, from the number of records identified in the search to the number of studies included in the review, ideally using a flow diagram.	4
	16b	Cite studies that might appear to meet the inclusion criteria, but which were excluded, and explain why they were excluded.	4
Study characteristics	17	Cite each included study and present its characteristics.	4,5
Risk of bias in studies	18	Present assessments of risk of bias for each included study.	4
Results of individual studies	19	For all outcomes, present, for each study: (a) summary statistics for each group (where appropriate) and (b) an effect estimate and its precision (e.g. confidence/credible interval), ideally using structured tables or plots.	5
Results of syntheses	20a	For each synthesis, briefly summarise the characteristics and risk of bias among contributing studies.	5-11
	20b	Present results of all statistical syntheses conducted. If meta-analysis was done, present for each the summary estimate and its precision (e.g. confidence/credible interval) and measures of statistical heterogeneity. If comparing groups, describe the direction of the effect.	5-11
	20c	Present results of all investigations of possible causes of heterogeneity among study results.	5-11
	20d	Present results of all sensitivity analyses conducted to assess the robustness of the synthesized results.	5-11
Reporting biases	21	Present assessments of risk of bias due to missing results (arising from reporting biases) for each synthesis assessed.	5-11
Certainty of evidence	22	Present assessments of certainty (or confidence) in the body of evidence for each outcome assessed.	4
**DISCUSSION**			
Discussion	23a	Provide a general interpretation of the results in the context of other evidence.	11,12
	23b	Discuss any limitations of the evidence included in the review.	12
	23c	Discuss any limitations of the review processes used.	12
	23d	Discuss implications of the results for practice, policy, and future research.	11-13
**OTHER INFORMATION**			

Section and Topic	Item #	Checklist item	Location where item is reported
Registration and protocol	24a	Provide registration information for the review, including register name and registration number, or state that the review was not registered.	2
	24b	Indicate where the review protocol can be accessed, or state that a protocol was not prepared.	2
	24c	Describe and explain any amendments to information provided at registration or in the protocol.	-
Support	25	Describe sources of financial or non-financial support for the review, and the role of the funders or sponsors in the review.	13
Competing interests	26	Declare any competing interests of review authors.	13
Availability of data, code and other materials	27	Report which of the following are publicly available and where they can be found: template data collection forms; data extracted from included studies; data used for all analyses; analytic code; any other materials used in the review.	13
